# Chiari 1 malformation and exome sequencing in 51 trios: the emerging role of rare missense variants in chromatin-remodeling genes

**DOI:** 10.1007/s00439-020-02231-6

**Published:** 2020-12-18

**Authors:** Aldesia Provenzano, Andrea La Barbera, Mirko Scagnet, Angelica Pagliazzi, Giovanna Traficante, Marilena Pantaleo, Lucia Tiberi, Debora Vergani, Nehir Edibe Kurtas, Silvia Guarducci, Sara Bargiacchi, Giulia Forzano, Rosangela Artuso, Viviana Palazzo, Ada Kura, Flavio Giordano, Daniele di Feo, Marzia Mortilla, Claudio De Filippi, Gianluca Mattei, Livia Garavelli, Betti Giusti, Lorenzo Genitori, Orsetta Zuffardi, Sabrina Giglio

**Affiliations:** 1 Medical Genetics Unit, Department of Clinical and Experimental Biomedical Sciences “Mario Serio”, University of Florence, Florence, Italy; 2 Department of Neurosurgery, “A. Meyer” Children Hospital of Florence, Florence, Italy; 3 Medical Genetics Unit, “A. Meyer” Children Hospital of Florence, Florence, Italy; 4 Department of Experimental and Clinical Medicine, Atherothrombotic Diseases Center, University of Florence, Careggi Hospital, Florence, Italy; 5 Department of Radiology, “A. Meyer” Children Hospital of Florence, Florence, Italy; 6 Department of Information Engineering, University of Florence, Florence, Italy; 7 Medical Genetics Unit, Department of Mother and Child, Azienda Unità Sanitaria Locale-IRCCS di Reggio Emilia, Reggio Emilia, Italy; 8 Unit of Medical Genetics, Department of Molecular Medicine, University of Pavia, Pavia, Italy

## Abstract

Type 1 Chiari malformation (C1M) is characterized by cerebellar tonsillar herniation of 3–5 mm or more, the frequency of which is presumably much higher than one in 1000 births, as previously believed. Its etiology remains undefined, although a genetic basis is strongly supported by C1M presence in numerous genetic syndromes associated with different genes. Whole-exome sequencing (WES) in 51 between isolated and syndromic pediatric cases and their relatives was performed after confirmation of the defect by brain magnetic resonance image (MRI). Moreover, in all the cases showing an inherited candidate variant, brain MRI was performed in both parents and not only in the carrier one to investigate whether the defect segregated with the variant. More than half of the variants were Missense and belonged to the same chromatin-remodeling genes whose protein truncation variants are associated with severe neurodevelopmental syndromes. In the remaining cases, variants have been detected in genes with a role in cranial bone sutures, microcephaly, neural tube defects, and RASopathy. This study shows that the frequency of C1M is widely underestimated, in fact many of the variants, in particular those in the chromatin-remodeling genes, were inherited from a parent with C1M, either asymptomatic or with mild symptoms. In addition, C1M is a Mendelian trait, in most cases inherited as dominant. Finally, we demonstrate that modifications of the genes that regulate chromatin architecture can cause localized anatomical alterations, with symptoms of varying degrees.

**Electronic supplementary material:**

The online version of this article (10.1007/s00439-020-02231-6) contains supplementary material, which is available to authorized users.

## Introduction

Primary or congenital malformations of Chiari are structural defects in the brain and spinal cord that occur during fetal development and range from cerebellar tonsillar herniation through the foramen magnum to the absence of the cerebellum with or without other associated intracranial or extracranial defects such as hydrocephalus, syrinx, encephalocele or spinal dysraphism. Four traditional subgroups of Chiari malformations (types CM 1–4) (Singh 2018; Holly 2019; Hidalgo 2020) are described despite the ambiguity between lumping and splitting makes hard the classification on clinical base (Luciano 2011; Hennekam 2012). The most common type 1 malformation (C1M) is characterized by the following magnetic resonance (MRI) findings: caudal displacement of the cerebellar tonsils more than 3–5 mm below the foramen magnum plane and a discrepancy between the anterior and posterior fossa and supratentorial crypt, with or without anomalies of the cranio-cervical junction (Bhimani 2018; de Arruda 2018; Lawrence 2018; Tam 2020). The signs of C1M can appear in adulthood or before adolescence, in the latter case with about 40% of subjects are under the age of 5 years, 25% of 5–10 years and 30% of 10–15 years (Tubbs 2007; McVige 2014; Piper 2019). The most common symptoms are occipital or upper cervical headaches, exacerbated by valsalva maneuver (tension, coughing, and sneezing) (McVige 2014; Piper 2019). Other symptoms vary greatly and may include motor (40–74%) and sensory (50%) changes in the extremities, clumsiness (15%), and dysphagia (10%) (Tubbs 2007; Piper 2019). C1M can also be associated with syringohydromyelia, hydrocephalus and several other malformations of the skull and cervical spine (Speer 2003; McVige 2014; Mukherjee 2019). Syringomyelia is present in 60–70% of patients, with progressive scoliosis in 30% of cases (Poretti 2016).

The presence of C1M in multiple members of the same family and not only in monozygotic twins strongly indicates a genetic basis for the malformation, even in the absence of Mendelian inheritance (Abbott 2018). Moreover, the clinical diagnosis is further complicated by the wide variety of symptoms and disease severity among members of the same family, some of them showing very nuanced features that do not significantly influence the daily routine (Abbott 2018). Under these conditions, the request for MRI investigations, essential for the diagnosis of C1M, is not always guaranteed.

Apart from these considerations, a very likely genetic basis for C1M is strongly supported by its presence in numerous genetic syndromes associated with different genes (Markunas 2013; Merello 2017; Rymer 2019; Martirosyan 2020). Although various information has emerged about the correlations between genetic variants and the presence of either isolated or syndromic C1M, a systematic genome-wide investigation with the extension to apparently healthy parents has never been conducted.

The aim of this study was to discover and validate C1M-associated genes by whole-exome sequencing (WES) in 51 among multiplex and single pediatric cases and in their relatives. Twenty-two patients were syndromic and 29 with isolated C1M (Table 1). In addition, we systematically extended MRI to both parents, regardless of whether they had clinical signs of C1M, in all the cases where the candidate variant(s) of the proband was inherited. To prevent that the interpretation of the MRI was influenced by the genetic data, the radiologist was not aware of which parent was the carrier of the candidate variant. Under these conditions, even the slightest sign of C1M in the carrier parent reinforced the role of the variant. In contrast, the parents of cases with de novo variants did not undergo MRI. In fact, all but one of these patients was syndromic, and the detected variant indeed fully accounted, at least a posteriori, for the clinical condition. This finding bona fide excluded the presence of other variant associated with the proband’s malformation in the healthy parents.

**Table 1:** Clinical and molecular findings of whole-exome sequencing/CGH array analysis of syndromic and isolated C1M cases

Case	Age at diagnosis	Sex	Clinical signs associated to C1M							Other clinical features	Pathogenic mutations	Category	Syndrome OMIM (#)	rs Id gnomAD NFE	Father		Mother	
			Syringomyelia	Bulbar kinking	Craniosynostosis	Hydrocephalus	Headache	Scoliosis	Ataxia						Molecular findings	S&S of C1M	Molecular findings	S&S of C1M
(a) Gene/genomic variants detected in patients with syndromes in which C1M was already reported																		
Case 1	8 years	F	Yes	No	–	–	–	Yes	–	Macrocephaly, ID, expressive language delay, aberrant right subclavian artery, atrial septal defect	5q35.2q35.3 (175,437,847–177,422,760) × 1	LoF	Sotos syndrome 1 (#117550)	–	WT	**–/–**	WT	**–/–**
Case 2	16 years	F	Yes	No	–	–	Yes	No	–	Macrocephaly, ID, behavioral problems, expressive language delay	*NSD1* c.[1090 T > C];[ =] p.[Tyr364His];[ =]	Missense	Sotos syndrome 1 (#117550)	N.A.	WT	**–/–**	WT	**–/–**
Case 3	5 years	M	Yes	No	Plagiocephaly	–	Yes	–	–	Microcephaly, Pierre Robin sequence, congenital heart defect (ventricular septal defect), inferior limb pain, sleep apnea, mild ID, short stature, T cell deficit	22q11(19,036,281–19,122,453) × 1	LoF	Chromosome 22q11.2 deletion syndrome (#611867)	–	WT	**–/–**	WT	**–/–**
Case 4	2 years	F	No	No	Complex craniosynostosis	Yes	Yes	–	Yes	global developmental delay, severe postnatal growth delay, hypotonia, torticollis, mandibular hypoplasia, dysmorphism, microcephaly, fifth finger clinodactyly	*PCNT* c.[7396G > A];[ =] p.[Glu2466Lys];[ =] c.[125A > C];[ =] p.[Lys42Thr];[ =] *ERF* c.[659C > A];[=] p.[Pro220His];[=]	Missense Missense Missense	Microcephalic osteodysplastic dwarfism (#210720) Craniosynostosis (#600775)	rs1270124196 (0.0009%) N.A. rs200767779 (0.04%)	c.[7396G > A];[=] p.[Glu2466Lys];[=] WT WT	**–/–**	WT c.[125A > C];[=] p.[Lys42Thr];[=] c.[659C > A];[=] p.[Pro220His];[=]	**+/+**
Case 5	7 years	M	No	No	Scaphocephaly	–	–	–	–	Speech delay, mild ID	*ERF* c.[203delG];[=] p.[Gly68Alafs*8];[=]	LoF	Craniosynostosis (#600775)	N.A.	WT	**–/–**	WT	**–/–**
Case 6	7 years	M	No	No	–	No	Yes	–	No	Mild ID	*ERF* c.[1367C > T];[=] p.[Thr456Met];[=]	Missense	Craniosynostosis (#600775)	rs376964824 (0.004%)	WT	**–/–**	c.[1367C > T];[=] p.[Thr456Met];[=]	**±**
Case 7	20 months	F	Yes	No	–	No	Yes	–	No	Nasal glioma	*BRAF* c.[430G > T];[=] p.[Val144Leu]; =]	Missense	Cardiofaciocutaneous syndrome (#115150)	N.A.	WT	**–/–**	c.[430G > T];[=] p.[Val144Leu];[=]	**±**
Case 8	9 years	M	No	No	No	No	–	No	No	ID, language delay, short stature, hypertelorism, epicanthic folds, café-au-lait spots on the abdomen	*CBL* c.[1112A > G];[=] p.[Tyr371Cys];[=]#	GoF	Noonan syndrome-like disorder with or without juvenile myelomonocytic leukemia (#613563)	rs267606706	WT	**–/–**	WT	**–/–**
Case 9	5 years	M	Yes	No	No	No	Yes	–	–	Fatty filum	*FGFR3* c.[1474A > G];[=] p.[Ile492Val];[=]	Missense	Crouzon syndrome and acanthosis nigricans (#612247)	rs745385417 (0.0009%)	WT	**–/–**	c.[1474A > G];[=] p.[Ile492Val];[=]	**+/+**
Case 10	4 years	F	No	No	No	No	Yes	–	No	–	*FGFR1* c.[2267G > A];[=] p.[Arg756His];[=]	Missense	Trigonocephaly (#190440)	rs374473310 (0.003%)	c.[2267G > A];[=] p.[Arg756His];[=]	±	WT	**–/–**
Case 11	4 years	F	No	No	–	–	Yes	–	–	Hypothyroidism, ventricular septal defect, growth delay, multiple congenital anomalies, short stature	*GNAS* c.[-168A > T];[=] p.[Met1ext-56];[=] *SETD2* c.[1775C > A];[=] p.[Thr592Lys];[=]	LoF Missense	Pseudohypoparathyroidism (#612463) Luscan–Lumish syndrome (#616831)	N.A. rs115569620 (0.1%)	WT c.[1775C > A];[=] p.[Thr592Lys];[=]	**+/+**	c.[-168A > T];[=]; p.[Met1ext-56];[=] WT	**–/–**

Case	Age at diagnosis	Sex	Clinical signs associated to C1M							Other clinical features	Pathogenic Mutations	Category	Syndrome OMIM (#)	rs Id gnomAD NFE	Father		Mother	
			Syringomyelia	Bulbar kinking	Craniosynostosis	Hydrocephalus	Headache	Scoliosis	Ataxia						Molecular findings	S&S of C1M	Molecular findings	S&S of C1M
(b) Gene/genomic variants detected in patients with syndromes in which C1M has never been reported																		
Case 12	8 years	M	No	No	No	No	Yes	–	No	Plagiocephaly, microcephaly, unusual movements, C3–C4 synostoses, hyperactivity, tics	*ZIC1* c.[493C > T];[=] p.[Leu165Phe];[=] *ZSWIM6* c.[2802C > G];[=] p.[Asp934Glu];[=]	Missense Missense	Coronal craniosynostosis (#616602) NEDMAGA syndrome (#617865)	rs761137004 (0.003%) rs1054922522 (0.005%)	c.[493C > T];[=] p.[Leu165Phe];[=] WT	**–/–**	WT c.[2802C > G];[=] p.[Asp934Glu];[=]	**–/–**
Case 13	10 years	F	Yes	Yes	–	–	Yes	No	–	Microphthalmia, nasal glioma, microcephaly, short stature, mild ID	*CENPE* c.[8080G > A];[=] p.[Asp2694Asn];[=] c.[304C > T];[=] p.[His102Tyr];[=]	Missense Missense	Microcephaly (#616051)	rs61756293 (0.1%) rs758681737 (0.02%)	c.[8080G > A];[=] p.[Asp2694Asn];[=] WT	**–/–**	WT c.[304C > T];[=] p.[His102Tyr];[=]	**–/–**
Case 14	13 years	F	No	No	–	–	Yes	No	–	Midfacial hypoplasia, GH deficiency, short stature, ID	*GLI2* c.[2977G > A];[=] p.[Val993Met];[=] c.[2117C > T];[=] p.[Thr706Ile];[=]	Missense Missense	Holoprosencephaly (#610829)	rs767022377 rs754899023 (0.0009%)	c.[2977G > A];[=] p.[Val993Met];[=] WT	**–/–**	WT c.[2117C > T];[=] p.[Thr706Ile];[=]	**+/+**
Case 15	12 years	F	Yes	No	No	No	Yes	No	Yes	Torticollis, pyramidal syndrome, tremor of a leg	*ALX4* c.[1036G > A];[=] p.[Val346Ile];[=] *TOR1A* c.[741delC];[=] p.[Asn247Lysfs];[=]	Missense LoF	Craniosynostosis (#615529) Torsional dystonia 1 syndrome (#128100)	rs751290539 (0.007%) N.A.	WT c.[741delC];[=] p.[Asn247Lysfs];[=]	**–/–**	c.[1036G > A];[=] p.[Val346Ile];[=] WT	**+/+**
Case 16	5 years	F	No	No	No	No	No	Yes	–	Microcephaly speech delay, hyperactivity, proteinuria, hypoplastic kidney, short stature	*EP300* c.[6627_6638delCCAGTTCCAGCA];[=] p.[Asn2209_Gln2213delinsLys];[=]^¤^	Inframe deletion	Rubinstein–Taybi syndrome (#613684)	rs587778256 (0.3%)	WT	**–/–**	WT	**–/–**
Case 17	4 years	F	No	No	–	–	Yes	–	–	Global developmental delay	*NALCN* c.[2870 T > C];[ =] p.[Met957Thr];[=] *SMYD3* c.[130C > T];[=] p.[Arg44stop];[=]	Missense LoF	CLIFAHDD syndrome (# 616,266)	N.A. rs772756510 (0.0035%)	WT WT	**–/–**	WT c.[130C > T];[=] p.[Arg44stop];[=]	**+/+**
Case 18	3 years	F	No	No	Trigonocephaly	–	Yes	–	–	Medulloblastoma	*PTCH1* c.[1538delA];[=] p.[Asp513Valfs];[=] *SMYD3* c.[152G > T];[=] p[.Arg51Leu];[=]	LoF Missense	Gorlin syndrome (#109400)	N.A. rs535565285	WT c.[152G > T];[=] p.[Arg51Leu];[=]	**+/+**	WT WT	**–/–**
Case 19	5 years	M	No	No	No	No	No	No	No	Verbal dyspraxia, speech delay, strabismus, hypermetropia, color blindness	*NSD3* c.[2098_2100delCTT];[=] p.[Lys700_700del];[=] *ACSL4* c.[77delT];[0] p.[Ile26fs];[0]	Inframe deletion LoF	X-linked mental retardation-63 (#300387)	rs1421928973 N.A.	WT WT	**–/–**	WT c.[77delT];[=] p.[Ile26fs];[=]	**–/–**
Case 20^1^	4 years	M	Yes	No	Scaphocephaly	–	Yes	–	–	ASD, psychomotor delay	*KDM5B* c.[293G > A];[ =] p.[Arg98His];[ =] *BCORL1* c.[3521A > G];[0] p.[ Asn1174Ser];[0]	Missense Missense	Autosomal recessive ID syndrome (#618109) Shukla–Vernon syndrome (#301029)	rs748231291 rs147703991 (0.007%)	WT WT	**–/–**	WT c.[3521A > G];[=] p.[Asn1174Ser];[=]	**–/–**
Case 21	2 years	F	No	No	–	–	No	–	–	Microcephaly, severe ID, hypotonia, seizures, brachydactyly, behavioral and sleep disturbances	*EHMT1* c.[3589C > T];[=] p.[Arg1197Trp];[=]§	LoF	Kleefstra syndrome (#610253)	rs137852727	WT	**–/–**	WT	**–/–**
Case 22	6 years	F	No	Yes	No	No	Yes	No	No	ID, bilateral foveal hypoplasia, strabismus and ptosis	*BRPF1* c.[330delC];[=] p.[Ile110fs];[=]	LoF	Intellectual developmental disorder with dysmorphic facies and ptosis (#617333)	N.A.	WT	**–/–**	WT	**–/–**

Case	Age at diagnosis	Sex	Clinical signs associated to C1M							Pathogenic mutations	Category	rs Id gnomAD NFE	Father		Mother	
			Syringomyelia	Bulbar kinking	Craniosynostosis	Hydrocephalus	Headache	Scoliosis	Ataxia				Molecular findings	S&S of C1M	Molecular findings	S&S of C1M
(c) Gene/genomic variants in isolated C1M																
Case 23	12 years	F	No	Yes	No	No	Yes	No	No	*SETD2* c.[5666 T > C];[=] p.[Met1889Thr];[=]	Missense	rs148097513 (0.1%)	c.[5666 T > C];[=] p.[Met1889Thr];[=]	**+/+**	WT	**–/–**
Case 24	8 years	M	No	No	No	No	Yes	No	No	*SETD2* c.[3388A > C];[=] p.[Lys1130Gln];[=] c.[557C > T];[=] p.[Pro186Leu];[=]	Missense Missense	rs1293115178 (0.0009%) rs78759480 (0.0065%)	c.[3388A > C];[=] p.[Lys1130Gln];[=] c.[557C > T];[=] p.[Pro186Leu];[=]	**+/+**	WT WT	**–/–**
Case 25	14 years	M	Yes	No	No	No	No	Yes	No	*SETD2* c.[6338G > A];[=] p.[Arg2113His];[=]	Missense	N.A.	c.[6338G > A];[=] p.[Arg2113His];[ =]	** + / + **	WT	**-/-**
Case 26	7 years	M	Yes	Yes	No	Yes (shunt)	No	No	No	*SETD2* c.[2188A > G];[=] p.[Lys730Glu];[=]	Missense	rs759927194 (0.0009%)	c.[2188A > G];[=] p.[Lys730Glu];[=]	** + / + **	WT	**–/–**
Case 27	4 years	M	Yes	No	No	No	Yes	No	No	*SETD1B* c.[23A > C];[=] p.[His8Pro];[=]	Missense	rs773794483 (0.2%)	WT	**–/–**	c.[23A > C];[ =] p.[His8Pro];[=]	**+/+**
Case 28	6 years	F	No	No	No	No	Yes	No	No	*SETD1B* c.[4333G > A];[=] p.[Ala1445Thr];[=]	Missense	rs144871159 (2%)	WT	**–/–**	c.[4333G > A];[=] p.[Ala1445Thr];[=]	**+/+**
Case 29	13 years	F	Yes	No	No	No	No	No	No	*SETD1B* c.[1100C > T];[=] p.[Pro367Leu];[=]	Missense	rs113279920 (0.6%)	c.[1100C > T];[=] p.[Pro367Leu];[=]	**+/+**	WT	**–/–**
Case 30	7 years	M	Yes	No	No	No	Yes	No	No	*SETDB1* c.[2657A > G];[=] p.[Lys886Arg];[=] *FGFR2* c.[1954A > G];[=] p.[Ile652Val];[=] *HIST1H1D* c.[94G > A];[=] p.[Gly32Arg];[=]	Missense Missense Missense	N.A. N.A. rs200822000 (0.03%)	WT WT c.[94G > A];[=] p.[Gly32Arg];[=]	**–/–**	c.[2657A > G];[=] p.[Lys886Arg];[=] c.[1954A > G];[=] p.[Ile652Val];[=] WT	**+/+**
Case 31	8 years	F	Yes	No	No	No	No	No	No	*HIST1H1E* c.[115C > T];[=] p.[Pro39Ser];[=] *HIST1H2BH* c.[11C > T];[=] p.[Pro4Leu];[=]	Missense Missense	rs567153678 rs868350265 (0.0009%)	WT c.[11C > T];[=] p.[Pro4Leu];[=]	**–/–**	c.[115C > T];[=] p.[Pro39Ser];[=] WT	**–/–**
Case 32	12 years	M	Yes	No	No	No	Yes	No	No	*SETBP1* c.[703C > T];[=] p.[Pro235Ser];[=]	Missense	rs751039254 (0.01%)	c.[703C > T];[=] p.[Pro235Ser];[=]	**+/+**	WT	**–/–**
Case 33	12 years	F	Yes (holocord)	No	No	No	No	No	No	*KDM6B* c.[752_753insACCACC];[752_753insACCACC] p.[Pro253_Pro254insLeu];[Pro253_Pro254insLeu]	Inframe insertion	rs771743551 (0.4%)	c.[752_753insACCACC];[=] p.[Pro253_Pro254insLeu];[=]	**–/–**	c.[752_753insACCACC];[=] p.[Pro253_Pro254insLeu];[=]	**–/–**
Case 34	3 months	F	No	No	Scaphocephaly	–	No	–	–	*KMT5A* c.[149G > A];[=] p.[Gly50Glu];[=]	Missense	N.A.	WT	**–/–**	WT	**–/–**
Case 35	21 years	F	No	Yes	Platybasia, mild scaphocephaly	No	Yes	No	No	*KMT2E* c.[4595G > A];[=] p.[Ser1532Asn];[=] c.[2722C > T];[=] p.[Pro908Ser];[=]	Missense Missense	rs74959149 (1%) rs151289792 (0.2%)	c.[4595G > A];[=] p.[Ser1532Asn];[=] WT	**–/–**	WT c.[2722C > T];[ =] p.[Pro908Ser];[=]	**–/–**
Case 36	11 months	F	No	No	No	No	No	No	No	*ALX4* c.[1162C > G];[=] p.[Arg388Gly];[=] *DKK1* c.[470G > T];[=] p.[Ser157Ile];[=]	Missense Missense	N.A. rs143388912 (0.1%)	WT WT	**–/–**	c.[1162C > G];[=] p.[Arg388Gly];[=] c.[470G > T];[=] p.[Ser157Ile];[=]	**+/+**
Case 37	2 years	F	Yes	Yes	No	No	No	No	Yes	*VANGL1* c.[1040A > C];[=] p.[Glu347Ala];[=]^d^	Missense	rs34059106 (0.01%)	c.[1040A > C];[=] p.[Glu347Ala];[=]	**+/+**	WT	**–/–**
Case 38	10 years	F	Yes	No	Mild trigonocephaly	No	Yes	Yes	No	*FUZ* c.[272C > T];[=] p.[Ser91Phe];[=]	Missense	rs750972427 (0.002%)	WT	**–/–**	c.[272C > T];[=] p.[Ser91Phe];[=]	**±**

Case	Age at diagnosis	Sex	Clinical signs associated to C1M							Pathogenic Mutations	Category	rs Id gnomAD NFE	Father		Mother	
			Syringomyelia	Bulbar kinking	Craniosynostosis	Hydrocephalus	Headache	Scoliosis	Ataxia				Molecular findings	S&S of C1M	Molecular findings	S&S of C1M
(d) Negative cases																
Case 39^2^	13 years	M	Yes	No	No	No	Yes	No	No	*SMYD4* c.[2390C > A];[2390C > A] p.[Pro797His];[Pro797His]	Missense	rs58337165 (0.06% in homo)	c.[2390C > A];[=] p.[Pro797His];[=]	**–/–**	c.[2390C > A];[=] p.[Pro797His];[=]	**–/–**
Case 40	3 years	M	No	No	No	No	Yes	No	No	*VANGL1* c.[346G > A];[346G > A] p.[Ala116Thr];[Ala116Thr]^d^	Missense	rs4839469 (1% in homo)	c.[346G > A];[=] p.[Ala116Thr];[=]	**–/–**	c.[346G > A];[=] p.[Ala116Thr];[=]	**–/–**
Case 41	14 years	F	No	No	No	No	Yes	No	No	Negative	–	–	Negative	NA	Negative	NA
Case 42	6 years	M	No	No	No	No	Yes	No	No	Negative	–	–	Negative	NA	Negative	NA
Case 43	11 years	M	No	No	–	–	Yes	No	–	Negative	–	–	Negative	NA	Negative	NA
Case 44	9 years	F	No	No	–	–	Yes	No	–	Negative	–	–	Negative	NA	Negative	NA
Case 45	13 years	M	No	Yes	–	–	Yes	No	–	Negative	–	–	Negative	NA	Negative	NA
Case 46	14 years	F	No	No	No	No	Yes	No	No	Negative	–	–	Negative	NA	Negative	NA
Case 47	9 years	F	No	No	No	No	Yes	No	No	Negative	–	–	Negative	NA	Negative	NA
Case 48	5 years	F	Yes	No	–	–	Yes	No	–	–	–	–	Negative	NA	Negative	NA
Case 49	10 years	M	Yes	No	No	No	Yes	No	No	Negative	–	–	Negative	NA	Negative	NA
Case 50	18 years	F	No	No	No	No	No	Yes	No	Negative	–	–	Negative	NA	Negative	NA
Case 51	5 years	M	Yes	No	No	No	No	No	No	Negative	–	–	Negative	NA	Negative	NA

*ASD* autism spectrum disorder, *GH* growth hormone, *CRMO* Chronic recurrent multifocal osteomyelitis, *ID* intellectual disability, *NFE* Non-Finnish European, *Ref* Reference, *GoF* Gain of function, *LoF* Loss of function, *AFR* African, *WT* wild type, *N.A.* not available, *C1M* Chiari Malformation type 1, homo homozygous. 1—case 20 has a brother that harbors the same *BCORL1* hemizygous variant and is affected by autism and macrocephaly. 2—case 39 has a brother that harbors the same *SMYD4* homozygous variant and is affected by C1M

S&S (signs and symptoms) of C1M: + MRI positive for C1M (tonsils herniation)/ + : recurrent headache or other symptoms of C1M; *NA* not available

+ MRI positive for C1M (tonsils herniation)/−: no symptoms of C1M

− MRI negative for C1M (tonsils herniation)/–: no symptoms of C1M

Identical variant detected by: ^#^Loh et al. (2009), ^¤^López et al. (2018), ^§^Yamada et al. 2018, ^d^Cai et al. (2014)

## Materials and methods

### Patients

The aim of the study was highlighting the molecular basis of C1M. To this purpose, we enrolled patients with either isolated or syndromic C1M. All patients have been evaluated by experts of neurosurgery, radiology as well as clinical genetics. Patients had a confirmed diagnosis of C1M on the following criteria: (a) caudal displacement of the cerebellar tonsils between 3 and 5 mm to the plane of the foramen magnum; (b) bulbar kinking (cases 13, 22, 23, 26, 35, 37, and 45; 13.7% of cases; (c) Klippel–Feil deformity (case 51; 1.96% of cases); (d) scoliosis (cases 1, 16, 25, 38, and 50; 9.8% of cases); (f) hydrocephalus (cases 4 and 26; 3.9% of cases). A measurement protocol defining the type of CM was followed upon classical guidelines recommendations (Lawrence 2018; Hidalgo 2020).

**Fig. 1:**
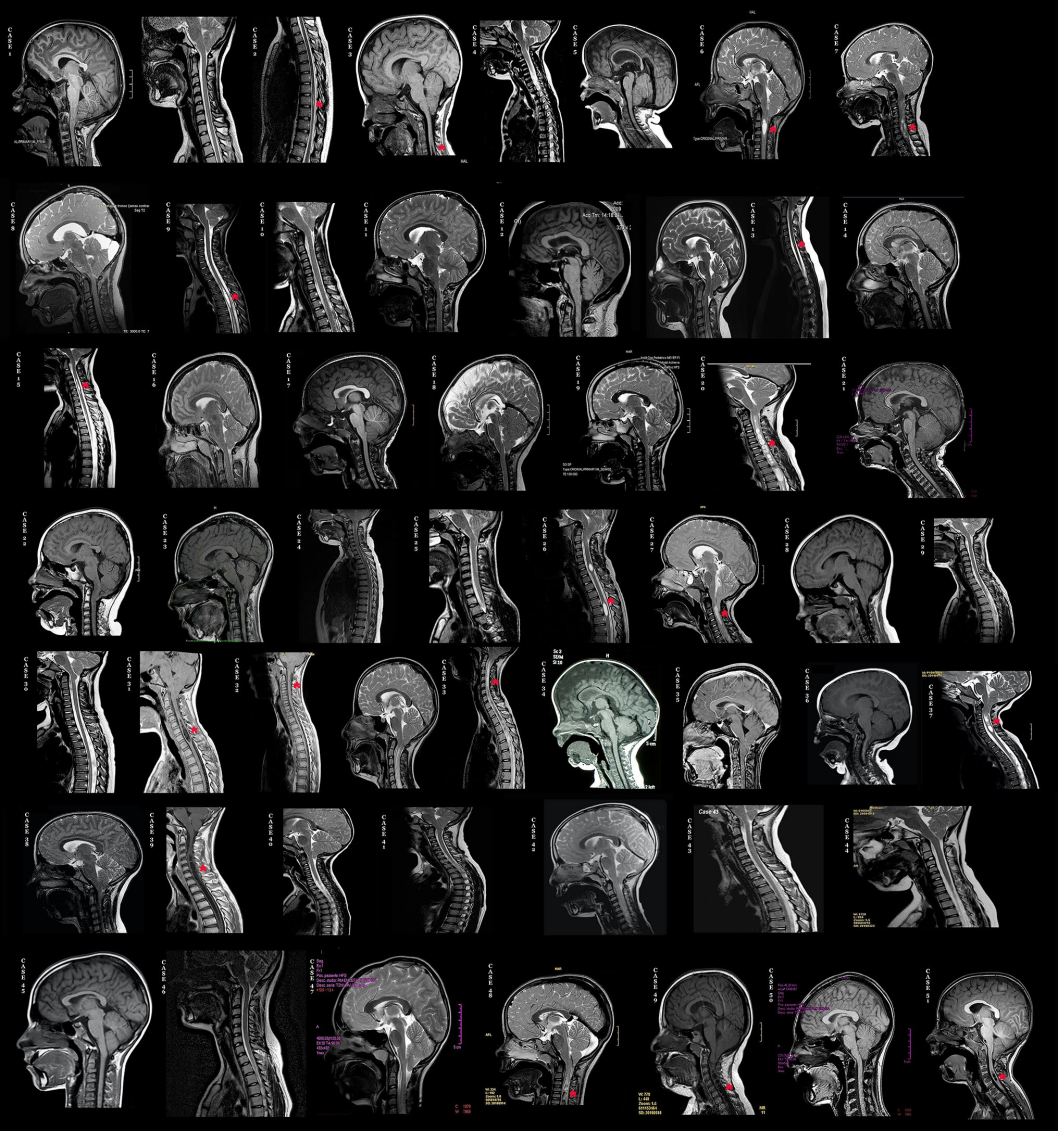
Sagittal T1 or T2-weighted MRI images showing Chiari type I malformation (C1M) in our 51 cases as evidenced by low-lying cerebellar tonsils. Sagittal MRI of the cervical spine also detects different cases of syringomyelia indicated by red asterisks

Patients were enrolled by the neurosurgeons who planned the type of operation, based on the clinical and radiological presentation of the Chiari Malformation (Fig. 1).

A written informed consent to proceed with genetic investigations was signed by the parents, according to the format of the Meyer Hospital. Samples were rendered anonymous, and each one marked with a progressive numerical code.

A total of 51 unrelated subjects (aged 3 months to 21 years, mean age 8 years) were enrolled. Chiari’s malformation appeared sporadic in all but four cases (cases 23, 25, 28, and 39) where C1M was already known to be familiar. The parents were regularly examined both from a clinical point of view and genetic investigations. The trio/quartet-based WES was performed together with the array-CGH analysis to rule out unbalanced genomic rearrangements. In 22 patients, other disorders, such as short stature, intellectual disability (ID), autism spectrum disorder (ASD), developmental delay, congenital anomalies or dysmorphic traits, were present in addition to C1M (Table 1). The clinical characteristics of the 51 probands including their age at diagnosis and the molecular findings are summarized in Table 1. More detailed information regarding recruiting and phenotype of the patients are reported in supplementary methods.

### Array-CGH

Array-CGH analysis was performed on proband’s DNA from blood using an Agilent Human Genome CGH Microarray Kit 4 × 180 k with an overall median probe space of 40 Kb, using the same protocol previously described (Palazzo 2017). All nucleotide positions refer to the Human Genome, February 2009 Assembly (hg19). Data analyses were performed using Agilent Cytogenomics V.2.5.8.1.

### Whole-exome sequencing (WES) and bioinformatics analysis

Genomic DNA (gDNA) was extracted according to the manufacturer’s instructions whereas libraries preparation for WES, sequencing base calling, coverage analysis, variants annotation, and variant filtering are detailed in the Supplementary Methods. MutationTaster, Mutation Assessor, SIFT, PolyPhen2_HVAR, FATHMM-MKL, and FATHMM were used to assess the effect of variants on protein function. Validated variants were classified based on standards and guidelines of the American College of Medical Genetics and Genomics (ACMG) (Nykamp 2017).

Quality control of sequencing showed that 96% of the reads were mapped to the reference genome (hg19), and 97% of the targeted regions were covered by ≧ 30 × reads with average depth of 120x. Details of quality control of depth, coverage, as well as the bioinformatically prioritized variant(s) and their interpretation are shown in Supplementary Methods and Supplementary Fig. 1).

All candidate variants were confirmed by Sanger sequencing.

### Total RNA extraction and gene expression profiling by Affymetrix GeneChip analysis

Skin biopsy samples were obtained from three probands and their family members and primary fibroblasts grown from skin biopsy specimens, as already reported (Vangipuram 2013). Total RNAs were extracted from confluent fibroblasts by RNeasy Mini Kit (Qiagen). The quality and quantity of extracted RNAs were assessed by Agilent 2100 Bioanalyzer System. Human Transcriptome Array 2.0 GeneChips were used. Further details are provided in Supplementary Methods.

## Results

Table 1 summarizes the main clinical findings of our C1M patients. Molecular results (array-CGH and WES) are also reported. According to the WES results, patients were divided into four categories (a–d): (i) affected by a syndrome in which C1M was already reported at least in some cases (a); (ii) affected by a syndrome in which C1M was never reported (b); (iii) affected by isolated C1M (c); and (iv) without any obvious disease-variant (d). Molecular findings in parents, their MRI findings (+: presence of C1M; −: absence of C1M), and their clinical condition in respect to C1M (presence at least of headache) are also reported. Table 1 also shows the frequency of the variant in gnomAD and/or its rs. In 11 cases, more than 1 gene showed candidate variants.

Category A (cases 1–11). Patients in this category have variants in genes previously reported in syndromes occasionally associated with C1M. With the exception of cases 7, 9 and 10, the others were with syndromic C1M, including developmental and psychomotor retardation. Although in all cases, the set of malformative signs was a posteriori attributable to the syndrome associated with the identified genetic/genomic variant, the clinical diagnosis was not a priori obvious. Brain MRI was performed due to frequent headache episodes in cases 9 and 10, and to a nasal glioma in case 7. Cases 9 and 10, both with a variant within an *FGFR* gene (*FGFR3* and *FGFR1*, respectively) did not show other clinical disorders except C1M. In cases 4, 6, 7, 9, 10, and 11, the imputed C1M-variant of the proband was inherited by a parent who was affected by recurrent headaches, whereas in the remaining cases was de novo. Analysis of the array-CGH experiments revealed only two cases (cases 1 and 3) with causative CNVs, namely deletion at 5q35, including *NSD1*, and 22q11.2, respectively. As expected by the syndromic condition of the patients, both were de novo.

Category B (cases 12–22). Patients in this category have variants in genes reported in syndromes in which C1M has never been described. All patients, but case 18 with trigonocephaly only, presented with malformative and/or behavioral characteristics not a priori ascribable to a specific syndrome. In all, MRI was requested because of neurological disorders and/or abnormal cranial conformation. The molecular and clinical conditions of the parents are described as for category A.

Category C (cases 23–38). Patients in this category have isolated C1M, sometimes associated with other individual signs or symptoms which, as a whole, were not attributable to any known syndrome. All candidate genes we detected have been reported in malformation syndromes that did not include C1M. MRI was requested in most of the cases because of recurrent headache while in cases 31, 33, 34, and 36 the investigation was requested because of motor tics, persistent arm pain, mild neurodevelopmental delay, and torticollis, respectively. Parents’ molecular and clinical conditions are described as above.

In category D (cases 39–51), three types of cases are listed: (i) a single case (case 39) in which the variant concerned a gene so far not disease-associated but whose segregation in the family coincided with the presence of C1M, thus strengthening the role of the gene in the Chiari malformation; (ii) a single case (case 40) in which the gene-variant had been reported in the literature with a disorder having a type of inheritance different from that found in our study; (iii) those in which no plausible variant has been identified (cases 41–51).

## Discussion

This study shows that C1M, both syndromic and isolated, is mainly dependent on variants in chromatin-remodeling genes, in most cases of the missense type and in very few cases presumably leading to a truncated protein (PTV protein truncation variant). Indeed, 25 out of the 45 variants lay in genes that play a direct role in maintaining chromatin organization (Table 2), while the remaining ones were in genes known for their role in bone and cartilage formation and bone fusion, and in neural tube defects. In 10 cases, WES and array investigations fail to reveal candidate variants (Table 1, d). These cases are with isolated C1M with recurrent headache and syringomyelia in three cases. The reason we have not identified any causative variant is attributable to different causes: (i) the variant has a frequency > 2% which is the maximum value of the MAF with which we have selected the variants; (ii) the variant is smaller than the CNVs identifiable by array-CGH and is found in regions covered < 20× reads by exome or is in non-coding regions; (iii) the variant is in mosaic with little or no percentage of variant allele in the blood. On the basis of what is reported in the literature, these last two hypotheses are the most probable (Kremer 2017; Wright 2019).

**Table 2:** Chromatin-remodeling proteins, nomenclature and substrate specificity

Histone methyltransferases				
Histone and residue	me3	me2	me1	
H3 lysine-36	NSD3 SETD2*	NSD1* NSD3 SETD2*	NSD1* SETD2*	Cases 1, 2 Case 19 Cases 11, 23, 24, 24, 26
H3 lysine-4	SMYD3* SETD1B*	SMYD3 * NSD3 SETD1B* KMT2E*	SETD1B* KMT2E*	Cases 17, 18 Case 19 Cases 27, 28, 29 Case 35
H3 lysine-9	SETDB1* EHMT1*	SETDB1* EHMT1*	SETDB1*	Case 30 Case 21
H3 lysine-27		NSD3		Case 19
H4 lysine-20			KMT5A*	Case 34

Histone demethylases				
Histone and residue	me3	me2	me1	
H3 lysine-4	KDM5B*	KDM5B*	KDM5B*	Case 20
H3 lysine-27	KDM6B#	KDM6B#		Case 33
Histone acetyltransferase				
Histone acetyltransferase activity	EP300*	Acetylates all four core histones in nucleosomes		Case 16
Histone H3 acetyltransferase activity	BRPF1*	Stimulates acetyltransferase and transcriptional activity		Case 22
Histone methylation				
DNA-binding protein	SETBP1*	Bind the SET nuclear oncogene which is involved in DNA replication		Case 32
Histone proteins				
Histone H1	HIST1H1D* HIST1H1E* HIST1H2BH*	Bind to linker DNA between nucleosomes forming the chromatin fiber		Case 30 Case 31 Case 31

*me1* mono-methylation, *me2* di-methylation, *me3* tri-methylation

*Heterozygous

^#^Biallelic

In the following sessions, we discuss the role played to the occurrence of C1M by chromatin-remodeling genes (category W), according to the different classes of coded proteins, from W1 to W5, and subsequently by the genes involved in the sutures of the cranial bones and in microcephaly (category X), in the closure of the neural tube (category Y), and in RASopathy (category Z).

### W. Chromatin-remodeling genes

Chromatin remodeling plays a central role in modulating gene expression. Histone post-translational modifications (PTMs) by specific enzymes, e.g., histone acetyltransferases (HATs), deacetylases, methyltransferases, and kinases, are crucial to chromatin dynamism. Although acquired alterations in the patterns of histone PTMs have been at first linked to cancer (Garraway 2013; Morgan 2015), the application of WES to cohorts of people with intellectual disabilities and other neurological dysfunction has shown that a growing number of constitutional disorders associate with germline variants of genes involved in chromatin regulation. In many cases, these genes are the same whose somatic variants, sometimes even the same variant, drive cancer. The resulting effect can be a constitutional disorder associated with higher than normal risk of tumors (Lee 2007; Acuna-Hidalgo 2017; Faundes 2018). More recently, new technological approaches for the efficient identification of DNA methylation throughout the genome have shown that syndromes caused by variants of the chromatin-remodeling genes are characterized by specific methylation signatures. These “episignatures”, typified either by hyper- or hypomethylation of the CpG sites, relate 42 clinically distinct OMIM disorders to 34 methylation patterns, potentially offering new therapeutic perspectives for individuals suffering from these disorders (Aref-Eshghi 2020). Many of the chromatin-remodeling genes associated with these syndromes, and in other contexts with cancer, are the same we have identified starting from a well-defined symptom such as C1M, although, paradoxically, C1M has been reported only occasionally or has never been described among the malformative characteristics that typify the various syndromes. The identified genes regarding chromatin-remodeling factors are listed in Table 2, based on their frequency in our cohort.

### W1. Histone methyltransferases

The most frequent variants associated with C1M were detected in members containing the SET domain of the lysine methyltransferase family. *SETD2* variants, all of missense type, were the most represented in our cohort (cases 11 and 23–26). Heterozygous variants of *SETD2* resulting in a premature stop codon have been associated with the Luscan–Lumish syndrome (#616831). Among the few subjects so far reported, some were with an overgrowth-Sotos-like phenotype and postnatal obesity (Lumish 2015; van Rij 2018; Marzin 2019). C1M with syringomyelia beside to neurodevelopmental disorders was reported in a single case with a *SETD2* frameshift variant (31). In contrast, none of our C1M patients with *SETD2* variants (Table 1 and Supplementary Fig. 2) were with Luscan–Lumish features although the 12-year-old patient (case 23) was beginning to show overweight. Patient 11, a 4-year-old girl, was the only syndromic one, although her clinical disorder was presumably related to the presence of the promoter *GNAS* variant inherited by the mother (Haldeman-Englert 2017). The fact that patients 23–26, aged between 7 and 14 years, were not in the least syndromic could be explained by the type of variant that in all cases was missense, and not a PTV one as in syndromic cases. In accordance with the relative pathogenicity of the missense variants, in all cases they were inherited from a parent who showed non-severe symptoms of C1M, essentially recurrent headaches. The consistency of paternal inheritance of the *SETD2* variants in our five cases might not be accidental. Maternal depletion of Setd2 causes defects in mouse oocyte maturation and subsequent one-cell arrest after fertilization, thus identifying *SETD2* as a crucial player in establishing the maternal epigenome and, in turn, embryonic development (Xu 2019). It is unclear why SETD2’s missense variants may specifically involve C1M.

Other frequent variants of histone methyltransferases, all of the missense type, have been detected in SETD1B. De novo variants in SETD1B, leading to PTV or haploinsufficiency, were reported in syndromic ID (Hiraide 2018; Krzyzewska 2019) (Supplementary Fig. 3). Our patients 27–29, aged between 4 and 13 years, were with isolated C1M, even if patient 29, the oldest one, was with mildly increased head circumference and dyslexia. All have inherited a missense variant from a parent with C1M, all with a history of recurring headaches. A specific hypermethylation signature associated with LoF variants in the *SETD1B* gene was recently demonstrated (Hunter 2005), with profiles overlapping those of patients affected by Hunter–McAlpine syndrome, who harbor duplication of *NSD1*. Indeed, *NSD1*, which also contains a SET domain, is responsible for Sotos1 syndrome mainly but not only in presence of frameshift and nonsense variants (https://varsome.com/gene/NSD1; Türkmen 2003; Laccetta 2017). Size variable microdeletions which encompass *NSD1* cause about 10% of Sotos1 syndrome cases, and, actually, C1M has been reported in few patients with this condition (Tatton-Brown 2005). Our cases 1 and 2, the first with a de novo 5q deletion of about 2 Mb including *NSD1,* and the second with a de novo rare missense variant in the same gene, showed C1M, accompanied in case 1 by syringomyelia. Both presented with a phenotype ascribable to Sotos1 syndrome (macrocephaly, ID, behavioral problems, and delay of the expressive language).

We also detected a rare variant in another gene of the NSD family, namely *NSD3*. The patient, case 19, is a syndromic 5-year-old child with visual problems (strabismus, hypermetropia, and color blindness) and ID. The presence of C1M was highlighted thanks to MRI that was performed to complete the instrumental exams. We discovered in the patient two variants, one inherited by the healthy mother, in the *ACSL4* gene associated with X-linked mental retardation-63 (#300387), which explains patient’s ID, and another one, a de novo inframe deletion in the *NSD3* gene. Its SET domain has lysine methyltransferase activity. The fact that the variant we detected is de novo and very rare reinforces its role in C1M. The three bases deletion falls into the C-terminal block of ~ 700 amino acids which is shared with NSD1, also associated with C1M (Supplementary Fig. 4).

Heterozygous variants in *SMYD3,* a gene that encodes a histone methyltransferase and so far involved in tumorigenesis only (Giakountis 2017; Bottino 2020), were detected in cases 17 and 18. Case 17 showed a stop variant, and case 18 a missense one, both inherited by highly symptomatic parents and both rare. In both cases, the phenotype was complicated by the presence of a de novo variant in a second gene, which perfectly matched with their clinical disorder. Indeed, case 17 with global developmental delay has a *NALCN* missense variant that matches with the CLIFAHDD syndrome (# 616266) overlapping patient’s phenotype, and case 18 was with a *PTCH1* frameshift variant associated with Gorlin syndrome (#109400). Actually, this patient who was enrolled because of trigonocephaly, 1 year later was with odontogenic keratocysts and developed a medulloblastoma, a tumor characteristic of Gorlin syndrome. Altogether, in both the cases, the rarity of the *SMYD3* variants and their segregation with C1M suggest their role in Chiari malformation. Of note, we detected a homozygous variant of *SMYD4,* an important paralogous of *SMYD3* (GeneCards: https://www.genecards.org/cgi-bin/carddisp.pl?gene=SMYD3) in two C1M brothers, inherited by healthy parents (case 39). However, the high frequency of this variant, also at the homozygous state (gnomAD), makes dubious its association with C1M in this family, unless assuming a complex inheritance.

All the other lysine-methyltransferase gene variants we detected in our cohort were in single cases, either with syndromic or isolated C1M. In case 34, a 3-month-old girl with scaphocephaly and delayed neurological development, we identified a de novo missense variant in *KMT5A* (or *SET8/PR-Set7)*. So far, no constitutional disturbances associated with variants of this gene have been identified. However, the finding that Kmt5a homozygous-null mice can only be recovered at 2.5 days post-conception (Oda 2009), might indicate that the paucity of *KMT5A*-associated disorders in humans is due to embryonic lethality. Indeed, the observed/expected (oe) metric (gnomAD) equal to 0.06 indicates that *KMT5A* is with high probability LoF intolerant. The variant we detected was absent in gnomAD although a very rare variant at the same location, p.Gly50Ala, is considered possibly damaging (Polyphen) or deleterious (SIFT). The finding that *KMT5A* interacts with *TWIST* in promoting epithelial–mesenchymal transition (Yang 2012), might explain patient 34 scaphocephaly. Indeed, null heterozygous variants of *TWIST* associates with unicoronal or bicoronal synostosis (Seto 2007).

In case 35, a 21-year-old female, we detected a compound heterozygosity for missense variants of *KMT2E* (Supplementary Fig. 5), another histone methyltransferase gene. Each of the variants was inherited from a healthy parent. De novo heterozygous variants of *KMT2E*, most predicted to result in PTV, are associated with O'Donnell-Luria–Rodan syndrome (#618512) characterized by global developmental delay with variably ID, and subtle dysmorphic features, not present in the patient. One of the variants we detected in the patient (c.[2722C > T];[=] p.[Pro908Ser];[=]) is low-frequent (0.001673), with two homozygous cases reported in gnomAD. The other one is absent in gnomAD although a very rare variant at the same location, p.Ser1532Arg, (0.000003995 of allele frequency) is given as deleterious at low confidence by SIFT. Patient 35 had a severe C1M, associated with platybasia and scaphocephaly and almost normal comprehension and behavioral skills, in the absence of any other clinical disorder. It is, therefore, logical to hypothesize that the situation of compound heterozygosity for *KMT2E* missense variants, a low frequent and a rare one, caused alterations only in a limited embryogenetic field, in this specific case the cranio–spinal junction. Indeed, there are numerous examples which demonstrate that the quantity of residual protein shapes the final phenotype, with rare biallelic LoF variants causing early lethality with aberration in numerous embryonic fields, and the combination of a rare and a low-frequent variant causing malformations limited to specific organs (Ren 2020).

In case 21, a 2-year-old girl with microcephaly, psychomotor delay, hypotonia, seizures, brachydactyly, behavioral and sleep disturbances, a de novo heterozygous variant of *EHMT1,* was detected. The gene codes a histone methyltransferase. The variant is not represented in gnomAD and is already reported as one of the few missense changes associated with Kleefstra syndrome, that is usually caused by large deletions, frameshift, or nonsense mutations. A posteriori, the malformative picture of our patient fits well with that of Kleefstra syndrome. The biochemical characterization of this variant, made on the previously reported patient, demonstrated that it deteriorates the histone methyltransferase-1 gene activity (Yamada 2018). It is noteworthy that in our patient the detection of the variant in a chromatin-remodeling gene, prompted us to investigate whether C1M, although never reported in Kleefstra syndrome, were present, as indeed it was the case (Fig. 1, case 21). Patients with Kleefstra syndrome are characterized by a specific alteration of the epimethylation signature, largely overlapping that of the Wiedemann–Steiner syndrome (WDSTS) (Aref-Eshghi 2020), associated with variants, mostly of truncating type, in *KMT2A*, encoding another lysine-methyltransferase. Indeed, the recent finding that WDSTS is almost always associated with C1M (Giangiobbe 2020) suggests that cranio-vertebral junction malformation can also be present in Kleefstra syndrome although so far neglect.

### W2. Histone demethylases

In contrast to histone methyltransferases, histone demethylases erase the methyl groups from histone lysine residues, A number of neurodevelopmental disorders, including both OMIM-codified and uncodified syndromes, are associated with heterozygous or hemizygous variants of the null type in histone demethylase genes (Swahari 2019). Moreover, as far as we know, C1M was never reported in alterations of any of histone demethylases.

In our C1M cohort, we detected variants in two histone demethylases, namely *KDM5B* (patient 20) and *KDM6B* (patient 33), (Supplementary Fig. 6). Case 20, a 4-year-old male with scaphocephaly, ASD and psychomotor delay, had two missense variants, one de novo in *KDM5B* and another inherited by the healthy mother in the X-linked gene *BCORL1.* The latter was also detected in his twin brother presenting with mild psychomotor delay. The patient was the only one in the family with C1M. Accordingly, we attributed the Chiari malformation to the variant in *KDM5B* whereas the one in *BCORL1* was presumably responsible for the mild neurodevelopmental disorder of both kids, as reported in Shukla–Vernon syndrome (#301029). *KDM5B*, also known as *JARID1B,* has been associated with an autosomal recessive ID syndrome (#618109), whereas a gain of function mechanism (GoF) has been suggested for the heterozygous missense variants detected in ID patients, (Lebrun 2018). In our case, the variant is de novo and falls within the BRIGHT domain, which contributes to the recognition of the H3K4me2 substrate peptide (Johansson 2016), that suggests its pathogenetic role (Supplementary Fig. 6a).

Case 33 is with homozygous variant in *KDM6B*, inherited by healthy parents, consisting in the duplication of two triplets, leading to three consecutive prolines outside from the functional domains but in a highly conserved region (Supplementary Fig. 6b). This variant should not impair the formation of the protein, as the gene constraint (gnomAD) indicates a strong probability of being LoF intolerant (oe score: 0.07). De novo heterozygous variants which are predicted protein truncating are associated with NEDCFSA (#618505), a neurodevelopmental disorder with coarse facies and mild distal skeletal abnormalities but apparently no C1M. In fact, our patient, with a totally different phenotype characterized only by C1M with severe holocord syrinx and mild ID, is homozygous for a variant that has no influence on heterozygous parents and, therefore, has a pathogenetic mechanism other than LoF.

### W3. Histone acetyltransferases

Histone acetylation and deacetylation are essential parts in optimizing gene regulation. In our cohort, we detected two C1M cases with variants in histone acetyltransferase genes, namely cases 16 and 22 and none with variants in those encoding histone deacetylases.

Case 16, a 5-year-old girl, has a unique sign evocative of C1M, namely scoliosis that is reported in up to 20% of patients with Chiari 1 malformation (Kelly 2015). No surgical treatment has been undertaken for C1M due to her severe psychomotor delay. The variant of this patient is an inframe de novo deletion in exon 31 of *EP300* which generates the loss of five amino acids, Asn-Gln-Phe-Gln-Gln with the insertion of Lys. This variant was considered pathogenic in a patient 42 in Lopez et al. (2018), presenting with a spectrum of signs fitting with Rubinstein–Taybi syndrome 2 (**#**613684, RSTS2). In fact, *EP300*, together with its paralogous *CREBBP*, is a gene whose variants are causative of Rubinstein Taybi syndrome by a mechanism of haploinsufficiency. Our patient’s evaluation by the clinical genetics’ team did not highlight the facial characteristics of RSTS, such as the typical grimacing smile, neither broad and/or angulated thumbs nor halluces. Rather, her features, with short stature, microcephaly, renal anomaly, and scoliosis, recalled the phenotype of the Menke–Hennekam syndrome 1 (#618332) associated with missense mutations in the distal exons of *CREBBP* (Banka 2019). The variant we found in patient 16 is a low-frequent one (0.3% among NFE, gnomAD), so that the assignment of a pathogenic role would require a second, hit that we were unable to identify even after exome reanalysis. Although C1M has been frequently reported in RBTS1, both for large deletions and for single-nucleotide variants regardless of their location, it has never been detected in variants associated with *EP300* (Parsley 2011; Marzuillo 2013; Menke 2018). Moreover, RSTS-associated variants in both *CREBBP* and *EP300* produce a specific epigenetic signature (Aref-Eshghi 2020).

Case 22, a 6-year-old girl with a de novo ultra-rare LoF variant in *BRPF1*, presents ID, bilateral foveal hypoplasia, strabismus and ptosis, a phenotype consistent with IDDDFP (#617333: intellectual development disorder with dysmorphic facies and ptosis), recently associated with the *BRPF1* heterozygous variants, mainly nonsense or frameshift (Mattioli 2017). Moreover, she suffered from recurrent headaches and MRI showed C1M with bulbar kinking (Fig. 1). Although brainstem hernia through the foramen magnum has never been reported in these patients, some were with C2–C3 spinal fusion of the cervical vertebrae. Indeed, malformations of this type, which fall within the spectrum of the Klippel–Feil anomaly, have been reported in cohorts of pediatric patients who attended the surgical treatment of symptomatic C1M (Tubbs 2003, 2011). *BRPF1* encodes a component of the MOZ/MORF which has a histone H3 acetyltransferase activity. The frameshift variant we detected should alter the interaction with KAT6A and KAT6B thus impairing *BRPF1* in activating KAT6A for H3K23 propionylation, as demonstrated for IDDDFP patients with variants comparable to that of patient 22 (Yan 2020). Not by chance, de novo truncating mutations in *KAT6B,* which cause a spectrum of disorders, include, although rarely, C1M (Kennedy 2019), and more frequently craniosynostosis which in turn may cause Chiari malformation (Bashir 2017).

### W4. DNA-binding proteins acting on histone methylation

Case 32, a 12-year-old boy with frequent headache episodes and hypospadias, had a missense variant in *SETBP1*, inherited from his father who was equally suffering from C1M and hypospadias. The SETBP1 protein is part of a group of proteins that binds to certain regions of DNA to increase gene expression. De novo missense germinal variants of *SETBP1* in a 12 base pair hotspot of exon 4, encoding SETBP1 protein residues 868–871, cause both Schinzel–Giedion syndrome (SGS) and hematological malignancies. In fact, the variants of this hotspot can alter a degron, thus compromising the correct degradation of the SETBP1 protein and leading to its accumulation (Piazza 2013, 2018).

In patient 32, the variant, also in exon 4, precedes the mutation hotspot for over 100 base pairs and is reported as very rare in gnomAD. Patient’s phenotype, decidedly less dramatic than that of SGS, could really depend on the *SETBP1* variant: missense variants upstream or downstream of the critical region can result in less severe disorders, hardly classifiable as SGS (see patients 28 and 29 in Acuna-Hidalgo 2017). Hypospadias, which is present in both our proband and his father, is actually reported in most of the SGS cases. As for C1M, it is never reported in SGS patients although skull malformation, wide occipital synchondrosis, steep base are frequently reported in subjects with C1M.

### W5. H1 linker histones

Histone H1 belongs to the fifth family of histones and binds to nucleosomes in several ways. Its interaction with the linker DNA strengthens the structure of the nucleosome, acting itself as histone linker (Fyodorov 2018). Like the core histones, H1 proteins undergo several post-translational modifications.

Case 31, an 8-year-old girl, had two variants in different classes of the linker histone H1 protein family, each inherited by a healthy parent. Due to her motor tics which she had suffered from since the age of 5 years, MRI was required leading to the detection of syringomyelia. None of the two genes, which lie in the large histone gene cluster on chromosome 6p22, have ever been associated with tics or Chiari malformation. Although the presence of tics has rarely been reported in C1M patients (Monzillo 2007; Berthet 2014), the two disorders are related as evidenced by the amelioration of symptoms after the surgical posterior fossa decompression, in both our and a similar case reported by Berthet et al. (Berthet 2014). One of them involves the *HIST1H2BH* gene, which currently is not associated with any of the OMIM-codified disorders, the other one involves the *HIST1H1E* gene whose de novo frameshift variants at the C-terminal tail associate with an ID syndrome, the Rahman Syndrome (#617537), characterized by a peculiar pattern of growth, specific facial features, premature aging and a specific hypomethylation profile (Ciolfi 2020). Patient 31 has no symptoms of Rahman syndrome, as expected by the location of the variant at the opposite end of the gene, i.e., in the N-terminal domain. The link between the two variants we detected, and the patient’s disorder is unclear. Both variants are extremely rare, which suggests their causality. On the other hand, the fact that the parents, each carrier of one of the variants, are healthy, suggests that it is precisely the co-presence of the two variants that determines their causality, thus suggesting some interactions between the two H1 linkers, either direct or mediated by common chaperone proteins (Flanagan 2016).

## X. Genes involved in the sutures of the cranial bones or microcephaly

In our study, ten patients (cases 4, 5, 6, 9, 10, 11, 14, 15, 30, and 36) were with variants in genes known to be associated with craniosynostosis. The premature fusion of the cranial sutures is a phenotypically and genetically heterogeneous condition, which is syndromic in most of the cases. Variants in *FGFR2*, *FGFR3*, *TWIST*, *EFNB1*, *TCF12* and *ERF* are detected but many other genes such as *FGFR1* and *ALX4* are emerging as crucial to a proper suture of cranial bones (Wilkie 2017).

Cases 4, 5, and 6 were with *ERF* heterozygous variants. *ERF* encodes a negative regulator of ERK1/2, at the base of the pathway RAS-MAP kinase; its haploinsufficiency can results in the failure to inhibit RUNX2 function (Twigg 2013). Variants in this gene are associated with syndromic (facial dysmorphism, speech delay, learning difficulties and/or behavioral problems as well as C1M) or non-syndromic forms of craniosynostosis (Glass 2019).

Case 4, a 2-year-old girl was with global developmental delay, severe postnatal growth delay, hypotonia, microcephaly, mandibular hypoplasia, fifth finger clinodactyly, ataxia with clumsy, and unstable gait. Beside to the *ERF* variant inherited by the mother, she also had biallelic missense variants in *PCNT*, inherited by each of the parents. The mother was complaining recurrent headaches. Accordingly, the *ERF* variant, a very rare one, was considered causative of the C1M disorder whereas the *PCNT* variants appeared to be responsible for the osteodysplastic primordial dwarphism type 2 (#210720), which may present also with microcephaly, and mandibular hypoplasia. Case 5, a 7-year-old boy, was with scaphocephaly, speech delay and mild ID. His ERF de novo variant was a frameshift one, so far never reported. Case 6, a 7-year-old boy, was with mild ID and recurrent headache. His missense and rare *ERF* variant was inherited by the mother, who resulted to be also with C1M but without any obvious symptom.

Three patients (cases 9, 10, and 30) were with variants in genes belonging to the *FGFR* gene family. The deregulation of the FGFR signaling network has been reported in different conditions, although the most well characterized are craniosynostosis and skeletal dysplasia (Miraoui 2010).

Case 9, a 5-year-old boy, was with syringomyelia, fatty filum, and headache. The missense variant in *FGFR3* was inherited by the mother who resulted to be affected by C1M and, indeed, she complained recurrent headache. The variant is very rare and falls in a transmembrane domain which is not involved in achondroplasia, craniosynostosis, or other skeletal FGFR3-associated dysplasia, although in some of these dysplasias Chiari malformations, of either type 1 or 2, are reported (Awad 2014). Moreover, a specific variant of *FGFR3*, p.Ala391Glu, that associates with Crouzon syndrome and acanthosis nigricans (CAN, #612247), is also characterized by C1M (Rymer 2019).

Case 10, a 4-year-old girl, was with recurrent headache. She has a missense variant in *FGFR1* that we assumed to be causative of C1M because it is very rare and was inherited by the father who resulted to be affected by C1M, although asymptomatic. *FGFR1* specific GoF mutations are associated with craniosynostosis in Pfeiffer and Jackson–Weiss syndromes, not present neither in the proband nor in the father. Chiari malformations are described in some of them (Sargar 2017).

Case 30, a 7-year-old boy with syringomyelia, mild ID and dizziness, was with three missense variants, one in *FGFR2*, a second one in *SETDB1*, and a third one in *HIST1H1D*, the first two inherited by the mother also with C1M, headache and dizziness, and the third inherited by the healthy father. The *FGFR2* variant seems to be mainly responsible for the C1M present in the proband and his mother. Indeed, variants in this gene have been associated with Chiari malformation in patients with craniosynostosis (Twigg 2015; Coll 2019).

Two patients (cases 15 and 36) were with missense variants in *ALX4* who is a homeodomain-like transcription factor. Heterozygous variants of this gene, leading to GoF and LoF, associate with craniosynostosis (#615529) and parietal foramina 2 (#609597), respectively, whereas biallelic variants are reported in frontonasal dysplasia (#613451).

Case 15, a 12-year-old girl, suffered from syringomyelia, stiff neck and pyramidal syndrome characterized by tremor in the legs, which started at the age of about 6 years. Of her two variants that we have detected, that in *ALX4*, a missense one, was inherited by the symptomatic C1M mother, while the *TOR1A* variant, a PTV one, was inherited by the father who, in retrospect, was affected by the same pyramidal syndrome of the proband, with strong general tremor since the age of 17 years diagnosed as juvenile Parkinson’s disease. Indeed, *TOR1A* variants are associated with dominant torsional dystonia 1 syndrome (DYT1, #128100).

Case 36, an 11-year-old girl, was with persistent congenital torticollis which suggested the MRI investigation. We found two missense variants in *ALX4* and *DKK1*, both inherited from the mother who suffered from recurrent headache. DKK1 is involved in the WNT-signaling cascade, with a role in the formation of the head during mouse embryogenesis (GeneCards, https://www.genecards.org/cgi-bin/carddisp.pl?gene=DKK1). Two different *DKK1* missense variants, both falling in domain 1, are reported in two familial cases of C1M (Merello 2017). The *DKK1* variant in our family, which falls between domains 1 and 2, supports its role in C1M, although it cannot be excluded that also the variant in *ALX4* has had a causative role.

The fact that both are inherited from the symptomatic mother (recurrent headache) supports their role, but does not allow us to understand which of the two (or if both) is involved in the Chiari malformation.

Case 12 showed two variants in two different genes, namely *ZIC1* (inherited from the father) and *ZSWIM6* (inherited from the mother). Heterozygous mutations in *ZIC1* are associated with coronal craniosynostosis-6 (CRS6-#616602) and some cases can have impaired ID with Dandy–Walker malformation as well as spina bifida occulta (Blank 2011). *ZIC1* GoF mutations are described in subjects with bicoronal synostosis and calvarial abnormality (plagiocephaly), with abnormal conformation of the posterior fossa associated with mild delayed development (Twigg 2015). Our case presented plagiocephaly, microcephaly, C3–C4 synostoses, clumsy movements, hyperactivity, and tics (successfully treated with Risperdal). Many of these features are described in patients with NEDMAGA condition (#617865) caused by heterozygous mutation in the *ZSWIM6* gene (Palmer 2017). At the moment, there are very few cases reported and the exact pathogenetic mechanism linked to variants in this protein is not well known. We assume that the neurological features of our case were determined by the variant in *ZSWIM6* while the *ZIC1* variant was associated with the defect of the posterior cranial fossa, in turn responsible for C1M, although a synergistic effect of the two variants was not excluded. In fact, none of the parents presented C1M, although the mother experienced difficulties with learning and a delay in language, presumably related to the *ZSWIM6* variant.

In case 13, WES revealed two compound heterozygous variants within the *CENPE* gene, inherited from healthy parents. This gene encodes for a centrosomal protein that accumulates in the G2 phase of the cell cycle and is required for stable spindle microtubule capture at kinetochores (GeneCard, https://www.genecards.org/cgi-bin/carddisp.pl?gene=CENPE). Diseases associated with CENPE include Microcephaly-13 (#616051). Our patient is a female of 10 years of age, with microphthalmia, microcephaly, short stature, ID and nasal glioma. Recent studies provided further knowledge about genes involved in microcephaly and few homozygous variants have been found in *CENPE* (Mirzaa 2014; Ahmad 2017). Furthermore, CENPE protein is highly expressed in some tumors including glioma (Rahane 2019) and indeed our case was with nasal glioma. Although our case is the first one in which biallelic variants in this gene are associated with C1M, we assume that the microcephaly and the associated posterior fossa malformation was the cause of C1M (Niesen 2002). In fact, the possibility that C1M is secondary to tumor formation as suggested (Kular 2020) cannot be formally ruled out although it seems unlikely since nasal glioma usually has no connection with the intracranial brain.

Case 14, a 13 year-old-girl with midfacial hypoplasia, GH deficiency, short stature and ID, presented biallelic missense variants in the *GLI2* gene. One of these is very rare (0.0009% of heterozygous people reported in gnomAD) and was inherited from her symptomatic mother (recurrent headache), while the other (inherited from the healthy father) has never been reported. GLI2 is a zinc-finger transcription factor involved in the Sonic Hedgehog pathway, and *GLI2* variants have been reported in holoprosencephaly (HPE) and/or holoprosencephaly-like (HPEL) phenotypes with pituitary anomalies and postaxial polydactyly (Bertolacini 2012). A link between HPE and craniosynostosis is reported as well as between craniosynostosis and C1M (Muenke 2001). To our knowledge, no biallelic inheritance has been reported for *GLI2*, but based on the segregation of the variants in our family, the maternal one [c.2117C > T] appears causative of C1M. At present, two different *GLI2* variants in cis, c.3351C > A and 3555delC have been reported in a patient with C1M and mild sign of HPE, inherited from an apparently normal mother (Arnhold 2015), indeed suggesting a role of *GLI2* in C1M.

## Y. Genes associated with neural tube defects (NTDs)

In three cases (37, 38, and 40), all with isolated C1M, we detected variants in two genes, namely *VANGL1*, which belongs to the planar cell polarity (PCP) core, in cases 37 and 40, and *FUZ*, a PCP effector gene, in case 38, both allowing coordination of cell movements during embryonic morphogenesis (Juriloff 2012). Rare germline heterozygous missense variants in both genes, as well as somatic ones in *VANGL1*, have been associated with isolated neural tube defects (Seo 2011; Tian 2020). Indeed, most of these variants behave as hypomorphic or conditional factors predisposing to the failure of the closure of the neural tube, as suggested by both their possible presence also in healthy relatives and the known contribution to the defect of nutritional factors such as folic acid (Bartsch 2012).

Although none of our patients was affected by NTD, a link between neural tube defects and craniospinal junction alteration is evident, since myelomeningocele, a type of NTD, is often associated to type II Chiari malformation with brainstem herniation and a towering cerebellum in addition to the herniated cerebellar tonsils and vermis (Kular 2020).

In patient 37, a 2-year-old girl with severe signs of Chiari I malformation such as sleep apnea, dysphagia, and ataxia with poor coordination and unsteady walk, the heterozygous missense variant of *VANGL1*, p.Glu347Ala, was inherited from the father who had C1M associated with recurrent headaches. The variant, interpreted as possibly damaging and deleterious by Polyphen and SIFT, respectively, is relatively frequent in the African population in which it is also present in the homozygous state, while it is extremely rare in other populations (0.0001238 among NFE to which the patient belongs: gnomAD). The variant is found in the carboxylic terminal of the protein, essential for its binding to the plasma membrane to make it available to the other PCP core proteins (Iliescu 2014). The functional study of an NTD variant that fell within the same domain (p.Arg374His) showed that the protein localized throughout the cytoplasm, rather than being enriched in the plasma membrane. It is quite conceivable that the occurrence of a defect in the closure of the neural tube rather than in the range of the C1M depends on the residual amount of protein still able to locate correctly on the cell membrane.

In patient 40, a 3-year-old-boy with severe stuttering, the *VANGL1* missense variant, p.Ala116Thr, was homozygous with heterozygous healthy parents. This allele, rs4839469, in its genotype GC (Alanine versus Proline) much more than in the GA one (Alanine versus Threonine), was associated with increased risk of NTDs in a Han population of Northern China (Cai 2014).

In conclusion, even if the world-wide high frequency of the variant makes dubious its role in C1M, we may assume *VANGL1* behaves as a susceptibility factor not only in NTDs but also in C1M occurrence.

Case 38, a 10-year-old girl with mild trigonocephaly and brachydactyly, has a missense variant in the *FUZ* gene, inherited by the mother who was also with an almost asymptomatic C1M. FUZ, is a PCP effector involved in cilium biogenesis, whose protein interacts, among others, with VANGL1 (https://www.uniprot.org/uniprot/Q9BT04). Similarly, missense variants of *FUZ* were reported in association with the susceptibility to all types of NTDs, including Arnold–Chiari malformation type II (Seo 2015).

However, FUZ has also a role in the general skeletal development as recognized in knockout mice (Gray 2009) and, recently, in a 24-week fetus where homozygosity for a LoF variant was associated with a long, narrow chest, moderately short ribs, short long bones, hypoplastic tibiae and extreme polydactyly of all four limbs, whereas the carrier parents were healthy (Zhang 2018). The missense variant of patient 38, which is evaluated as probably damaging (Polyphen) and deleterious (SIFT), is very rare (0.002%, gnomAD). Indeed, this observation and the presence of C1M in her mother, who carries the same variant, suggest the role of the *FUZ* variant in her disorder.

## Z. RASopathies

RASopathies are a group of syndromes caused by germline variants in genes that encode components or regulators of the Ras/mitogen-activated protein kinase (MAPK) pathway (Liao 2019). C1M is not typical of these syndromes, although it has been sometimes reported. We detected two cases (cases 7 and 8) with variants in RASopathy genes namely *BRAF* and *CBL*, respectively.

Case 7, a 20-month-old girl, presented with a missense variant in the *BRAF* gene, inherited from her asymptomatic mother, with C1M. *BRAF* heterozygous missense variants are associated with the Cardiofaciocutaneous syndrome (CFCS-#115150), characterized by cardiac abnormalities, distinctive craniofacial appearance, and cutaneous abnormalities. C1M has been reported in a number of cases (Rauen 2016).

The c.430G > T variant of our patient was not reported in the literature and, at the time of diagnosis, no clinical signs of CFCS were recognized, as in her mother. This variant falls into a domain associated with different types of tumors and indeed our patient was with nasal glioma (Turski 2016).

Case 8, a male of 9 years of age, showed ID, short stature, hypertelorism, epicanthal folds and café-au-lait spots; molecular analysis discovered a missense variant in the *CBL* gene (Tyr371Cys), that is known to be associated with Noonan syndrome (NS-#613563). Retrospectively, many signs could be attributed to the Noonan syndrome, albeit without the typical facial gestalt. Similarly, Martinelli et al. reported one case without the Noonan facial gestalt and C1M having a *CBL* variant located in the same domain of our case (Lys382Glu) (Martinelli 2010).

## Final conclusion

This study shows two main points:

1. C1M frequency is largely underestimated. Indeed, in 21 of our cases, the same variant of the C1M proband was detected in one parent who had an MRI positive for C1M. In most of the cases, the carrier parent showed signs of C1M, mainly recurrent headaches, which, however, were not a real impediment to an independent life lived without particular restriction. In fact, most of carrier parents were unaware of being affected by the same disorder of their kid. Although it is possible that with transition to adult life there is a physical and psychological adaptation to the damage of the malformation, the good health of the parent poses a warning to carry out invasive interventions. Of course, a different expression of the variant between parents and probands cannot be discarded.
2. C1M has a strong genetic basis, with Mendelian inheritance in most of the cases and a more complex inheritance in a few. The latter condition is essentially related to genes involved in NTDs, already known to result by a combination of multiple genes and environmental factors.

Genes associated with C1M are of two types: those directly associated with the development of skull and cranio-vertebral junction (listed in categories X, Y, and Z) and those involved in chromatin remodeling (category W). The impact of chromatin-remodeling genes on the formation of the cranio-vertebral junction is not clear. Episignatures of patients with syndromes where C1M has sometimes been reported show varying degrees of abnormal methylation (Aref-Eshghi 2020). For example, while SETD1B variants in the SETD1B-related syndrome result in a strong hypermethylated signature, NSD1 variants associated with Sotos syndrome display remarkable hypomethylation. In contrast, intermediate episignature alteration was detected in Wiedemann–Steiner syndrome, Kleefstra syndrome and Rubinstein–Taybi syndrome where C1M can be present. This indicates that the correct formation of the cranio-vertebral junction requires the interplay of multiple genes, each of them activated or silenced in a specific space–time span of embryogenesis.

In most of the cases, the gene variants in chromatin-remodeling disorders are de novo, heterozygous and protein truncating, suggesting that haploinsufficiency is the predominant mechanism for the associated diseases. In contrast, in our isolated C1M cases, we mainly detected inherited variants of missense and inframe type that do not seem to destabilize the entire protein function and do not jeopardize the survival of the embryo. Although we have not performed methylation tests and the global expression profile in fibroblasts from three probands and their carrier parent did not reveal any alteration (supplementary methods), we consider the chromatin-remodeling variants as causative of C1M because they (i) segregated with an affected parent, (ii) were at low frequency in contrast to the relative frequency of the C1M malformation, and (iii) always affected conserved domains and were considered pathogenic or likely pathogenic by the prediction tools.

In general, in our study, no associations emerged between recurrent malformations related to C1M and specific gene variants. In particular, there are no clinical signs that are pathognomonic of a particular gene/variant, apart from in cases with known syndromes. However, the individual cases with variants on the same gene are too few to identify any distinctive features. In addition, the surgery treatment resulted in radiological improvements of the C1M, regardless of the causative variant.

Our results also highlight that in syndromic cases, the presence of C1M was not an accidental finding but rather the effect of the variant in different tissues and timing of embryogenesis. This is clearly shown by the variants of NSD1 in cases 1 and 2, who were later assigned as affected by Sotos syndrome, and the variants identified in *ERF*, *FGFR1*, and *FGFR3*, all genes associated with craniosynostosis and, rather frequently, Chiari malformation.

This study also showed that there is still much space for the attribution of the causative gene(s) to non-severe and non-rare disorders. To this end, it becomes imperative to increase the numerousness of patients with that certain condition, extend molecular and clinical investigations to family members, and take into consideration also low-frequent, benign, or probably benign variants even if present in apparently healthy parents.

## Electronic supplementary material

Below is the link to the electronic supplementary material.

Supplementary file 1 (DOCX 43 kb)

Supplementary file 2 (TIF 101587 kb)

Supplementary file 3 (TIF 103 kb)

Supplementary file 4 (TIF 330 kb)

Supplementary file 5 (TIF 17518 kb)

Supplementary file 6 (TIF 307 kb)

Supplementary file 7 (TIF 25831 kb)

## References

[CR1] Abbott D, Brockmeyer D, Neklason DW (2018). Population-based description of familial clustering of Chiari malformation Type I. J Neurosurg.

[CR2] Acuna-Hidalgo R, Deriziotis P, Steehouwer M (2017). Overlapping SETBP1 gain-of-function mutations in Schinzel-Giedion syndrome and hematologic malignancies. PLoS Genet.

[CR3] Ahmad I, Baig SM, Abdulkareem AR (2017). Genetic heterogeneity in Pakistani microcephaly families revisited. Clin Genet.

[CR4] Al-W A, Aleck KA, Bhardwaj RD (2014). Concomitant achondroplasia and Chiari II malformation: a double-hit at the cervicomedullary junction. World J Clin Cases.

[CR5] Aref-Eshghi E, Kerkhof J, Pedro VP (2020). Evaluation of DNA Methylation episignatures for diagnosis and phenotype correlations in 42 Mendelian neurodevelopmental disorders. Am J Hum Genet.

[CR6] Arnhold IJ, França MM, Carvalho LR (2015). Role of GLI2 in hypopituitarism phenotype. J Mol Endocrinol.

[CR7] Banka S, Sayer R, Breen C (2019). Genotype-phenotype specificity in Menke–Hennekam syndrome caused by missense variants in Exon 30 or 31 of CREBBP. Am J Med Genet A.

[CR8] Bartsch O, Kirmes I, Thiede A (2012). Novel VANGL1 gene mutations in 144 Slovakian, Romanian and German patients with neural tube defects. Mol Syndromol.

[CR9] Bashir RA, Dixit A, Goedhart C (2017). Lin-Gettig syndrome: craniosynostosis expands the spectrum of the KAT6B related disorders. Am J Med Genet A.

[CR10] Berthet S, Crevier L, Deslandres C (2014). Abnormal movements associated with oropharyngeal dysfunction in a child with Chiari I malformation. BMC Pediatr.

[CR11] Bertolacini CD, Ribeiro-Bicudo LA, Petrin A (2012). Clinical findings in patients with GLI2 mutations–phenotypic variability. Clin Genet.

[CR12] Bhimani AD, Esfahani DR, Denyer S (2018). Adult Chiari I malformations: an analysis of surgical risk factors and complications using an International Database. World Neurosurg.

[CR13] Blank MC, Grinberg I, Aryee E (2011). Multiple developmental programs are altered by loss of Zic1 and Zic4 to cause Dandy-Walker malformation cerebellar pathogenesis. Development.

[CR14] Bottino C, Peserico A, Simone C (2020). SMYD3: An oncogenic driver targeting epigenetic regulation and signaling pathways. Cancers (Basel).

[CR15] Cai C, Shi O, Wang B (2014). Association between VANGL1 gene polymorphisms and neural tube defects. Neuropediatrics.

[CR16] Ciolfi A, Aref-Eshghi E, Pizzi S (2020). Frameshift mutations at the C-terminus of HIST1H1E result in a specific DNA hypomethylation signature. Clin Epigenetics.

[CR17] Coll G, Ouadih YE, Abed-Rabbo F (2019). Hydrocephalus and Chiari malformation pathophysiology in FGFR2-related faciocraniosynostosis: a review. Neurochirurgie.

[CR18] de Arruda JA, Figueiredo E, Monteiro JL (2018). Orofacial clinical features in Arnold Chiari type I malformation: a case series. J Clin Exp Dent.

[CR19] Faundes V, Newman WG, Bernardini L (2018). Histone lysine methylases and demethylases in the landscape of human developmental disorders. Am J Hum Genet.

[CR20] Flanagan TW, Brown DT (2016). Molecular dynamics of histone H1. Biochim Biophys Acta..

[CR21] Fyodorov DV, Zhou BR, Skoultchi AI (2018). Emerging roles of linker histones in regulating chromatin structure and function. Nat Rev Mol Cell Biol.

[CR22] Garraway LA, Lander ES (2013). Lessons from the cancer genome. Cell.

[CR23] Giakountis A, Moulos P, Sarris ME (2017). Smyd3-associated regulatory pathways in cancer. Semin Cancer Biol.

[CR24] Giangiobbe S, Caraffi SG, Ivanovski I (2020). Expanding the phenotype of Wiedemann-Steiner syndrome: craniovertebral junction anomalies. Am J Med Genet part A.

[CR25] Glass GE, O'Hara J, Canham N (2019). ERF-related craniosynostosis: the phenotypic and developmental profile of a new craniosynostosis syndrome. Am J Med Genet A.

[CR26] Gray RS, Abitua PB, Wlodarczyk BJ (2009). The planar cell polarity effector Fuz is essential for targeted membrane trafficking, ciliogenesis and mouse embryonic development. Nat Cell Biol.

[CR27] Haldeman-Englert CR, Hurst ACE, Levine MA et al (2017) Disorders of GNAS Inactivation. In: GeneReviews® [Internet]. Seattle (WA): University of Washington, Seattle; 1993–2020 29072892

[CR28] Hennekam RC, Biesecker LG (2012). Next-generation sequencing demands next-generation phenotyping. Hum Mutat..

[CR29] Hidalgo JA, Tork CA, Varacallo M. (2020) Arnold Chiari Malformation. Treasure Island (FL): StatPearls Publishing; https://www.ncbi.nlm.nih.gov/books/NBK430685/ Accessed Jan 2020 28613730

[CR30] Hiraide T, Nakashima M, Yamoto K (2018). De novo variants in SETD1B are associated with intellectual disability, epilepsy and autism. Hum Genet.

[CR31] Holly LT, Batzdorf U (2019). Chiari malformation and syringomyelia. J Neurosurg Spine.

[CR32] Hunter AG, Dupont B, McLaughlin M (2005). The Hunter-McAlpine syndrome results from duplication 5q35-qter. Clin Genet.

[CR33] Iliescu A, Gros P (2014). The intracellular carboxyl terminal domain of Vangl proteins contains plasma membrane targeting signals. Protein Sci.

[CR34] Johansson C, Velupillai S, Tumber A (2016). Structural analysis of human KDM5B guides histone demethylase inhibitor development. Nat Chem Biol.

[CR35] Juriloff DM, Harris MJ (2012). A consideration of the evidence that genetic defects in planar cell polarity contribute to the etiology of human neural tube defects. Birth Defects Res A Clin Mol Teratol.

[CR36] Kelly MP, Guillaume TJ, Lenke LG (2015). Spinal deformity associated with Chiari malformation. Neurosurg Clin N Am.

[CR37] Kennedy J, Goudie D, Blair E (2019). KAT6A syndrome: genotype-phenotype correlation in 76 patients with pathogenic KAT6A variants. Genet Med.

[CR38] Kremer LS, Bader DM, Mertes C (2017). Genetic diagnosis of Mendelian disorders via RNA sequencing. Nat Commun.

[CR39] Krzyzewska IM, Maas SM, Henneman P (2019). A genome-wide DNA methylation signature for SETD1B-related syndrome. Clin Epigenet.

[CR40] Kular S, Cascella M. (2020) Arnold Chiari Malformation. Treasure Island (FL): StatPearls Publishing; https://www.ncbi.nlm.nih.gov/books/NBK430685/ Accessed Jan 2020

[CR41] Laccetta G, Moscuzza F, Michelucci A (2017). A novel missense mutation of the NSD1 gene associated with overgrowth in three generations of an italian family: case report, differential diagnosis, and review of mutations of NSD1 gene in familial Sotos syndrome. Front Pediatr.

[CR42] Lawrence BJ, Urbizu A, Allen PA (2018). Cerebellar tonsil ectopia measurement in type I Chiari malformation patients show poor inter-operator reliability. Fluids Barriers CNS..

[CR43] Lebrun N, Mehler-Jacob C (2018). Novel KDM5B splice variants identified in patients with developmental disorders: functional consequences. Gene.

[CR44] Lee JH, Tate CM, You JS, Skalnik DG (2007). Identification and characterization of the human Set1B histone H3-Lys4 methyltransferase complex. J Biol Chem.

[CR45] Liao J, Mehta L (2019). Molecular genetics of Noonan syndrome and RASopathies. Pediatr Endocrinol Rev.

[CR46] Loh ML, Sakai DS, Flotho C (2009). Mutations in CBL occur frequently in juvenile myelomonocytic leukemia. Blood.

[CR47] López M, García-Oguiza A, Armstrong J (2018). Rubinstein-Taybi 2 associated to novel EP300 mutations: deepening the clinical and genetic spectrum. BMC Med Genet.

[CR48] Luciano MG (2011). Chiari malformation: are children little adults?. Neurol Res.

[CR49] Lumish HS, Wynn J, Devinsky O (2015). Brief Report: SETD2 mutation in a child with autism, intellectual disabilities and epilepsy. J Autism Dev Disord.

[CR50] Markunas CA, Soldano K, Dunlap K (2013). Stratified whole genome linkage analysis of Chiari type I malformation implicates known Klippel-Feil syndrome genes as putative disease candidates. PLoS ONE.

[CR51] Martinelli S, De Luca A, Stellacci E (2010). Heterozygous germline mutations in the CBL tumor-suppressor gene cause a Noonan syndrome-like phenotype. Am J Hum Genet.

[CR52] Martirosyan Z, Malhotra S (2020). Central sleep apnea and Chiari 1 malformation in a pediatric patient with Klippel-Feil syndrome. Clin Sleep Med.

[CR53] Marzin P, Rondeau S, Aldinger KA (2019). SETD2 related overgrowth syndrome: presentation of four new patients and review of the literature. Am J Med Genet C Semin Med Genet.

[CR54] Marzuillo P, Grandone A, Coppola R (2013). Novel cAMP binding protein-BP (CREBBP) mutation in a girl with Rubinstein-Taybi syndrome, GH deficiency, Arnold Chiari malformation and pituitary hypoplasia. BMC Med Genet.

[CR55] Mattioli F, Schaefer E, Magee A (2017). Mutations in histone acetylase modifier BRPF1 cause an autosomal-dominant form of intellectual disability with associated ptosis. Am J Hum Genet.

[CR56] McVige JW, Leonardo J (2014). Imaging of Chiari type I malformation and syringohydromyelia. Neurol Clin.

[CR57] Menke LA, Gardeitchik T, DDD study (2018). Further delineation of an entity caused by CREBBP and EP300 mutations but not resembling Rubinstein–Taybi syndrome. Am J Med Genet A..

[CR58] Merello E, Tattini L, Magi A (2017). Exome sequencing of two Italian pedigrees with non-isolated Chiari malformation type I reveals candidate genes for cranio-facial development. Eur J Hum Genet.

[CR59] Miraoui H, Marie PJ (2010). Fibroblast growth factor receptor signaling crosstalk in skeletogenesis. Sci Signal..

[CR60] Mirzaa GM, Vitre B, Carpenter G (2014). Mutations in CENPE define a novel kinetochore-centromeric mechanism for microcephalic primordial dwarfism. Hum Genet.

[CR61] Monzillo P, Nemoto P, Costa A (2007). Paroxysmal hemicrania-tic and Chiari I malformation: an unusual association. Cephalalgia.

[CR62] Morgan MA, Shilatifard A (2015). Chromatin signatures of cancer. Genes Dev.

[CR63] Muenke M, Wilkie AOM, Scriver CR, Beaudet AL, Sly WS, Valle D (2001). Craniosynostosis syndromes. The metabolic and molecular bases of inherited disease.

[CR64] Mukherjee S, Kalra N, Warren D (2019). Chiari I malformation and altered cerebrospinal fluid dynamics-the highs and the lows. Childs Nerv Syst.

[CR65] Niesen CE (2002). Malformations of the posterior fossa: current perspectives. Semin Pediatr Neurol.

[CR66] Nykamp K, Anderson M, Powers M (2017). Sherloc: a comprehensive refinement of the ACMG-AMP variant classification criteria. Genet Med.

[CR67] Oda H, Okamoto I, Murphy N (2009). Monomethylation of histone H4-lysine 20 is involved in chromosome structure and stability and is essential for mouse development. Mol Cell Biol.

[CR68] Palazzo V, Provenzano A, Becherucci F (2017). The genetic and clinical spectrum of a large cohort of patients with distal renal tubular acidosis. Kidney Int.

[CR69] Palmer EE, Kumar R, Gordon CT (2017). A Recurrent de novo nonsense variant in ZSWIM6 results in severe intellectual disability without frontonasal or limb malformations. Am J Hum Genet.

[CR70] Parsley L, Bellus G, Handler M (2011). Identical twin sisters with Rubinstein-Taybi syndrome associated with Chiari malformations and syrinx. Am J Med Genet A.

[CR71] Piazza R, Valletta S, Winkelmann N (2013). Recurrent SETBP1 mutations in atypical chronic myeloid leukemia. Nat Genet.

[CR72] Piazza R, Magistroni V, Redaelli S (2018). SETBP1 induces transcription of a network of development genes by acting as an epigenetic hub. Nat Commun.

[CR73] Piper RJ, Magdum SA (2019). Chiari 1 malformation and raised intracranial pressure. Childs Nerv Syst.

[CR74] Poretti A, Ashmawy R, Garzon-Muvdi T (2016). Chiari type 1 deformity in children: pathogenetic, clinical, neuroimaging, and management aspects. Neuropediatrics.

[CR75] Rahane CS, Kutzner A, Heese K (2019). A cancer tissue-specific FAM72 expression profile defines a novel glioblastoma multiform (GBM) gene-mutation signature. J Neurooncol.

[CR76] Rauen KA (2016). Cardiofaciocutaneous Syndrome. In: Adam MP, Ardinger HH, Pagon RA, et al. (eds). University of Washington, Seattle, pp 1993–2020 20301365

[CR77] Ren X, Yang N, Wu N (2020). Increased TBX6 gene dosages induce congenital cervical vertebral malformations in humans and mice. J Med Genet.

[CR78] Rymer K, Shiang R, Hsiung A (2019). Expanding the phenotype for the recurrent p.Ala391Glu variant in FGFR3: beyond crouzon syndrome and acanthosis nigricans. Mol Genet Genomic Med..

[CR79] Sargar KM, Singh AK, Kao SC (2017). Imaging of skeletal disorders caused by fibroblast growth factor receptor gene mutations. Radiographics.

[CR80] Seo JH, Zilber Y, Babayeva S, Liu J (2011). Mutations in the planar cell polarity gene, Fuzzy, are associated with neural tube defects in humans. Hum Mol Genet.

[CR81] Seo JH, Zilber Y, Babayeva S, Liu J (2015). Mutations in the planar cell polarity gene, Fuzzy, are associated with neural tube defects in humans. Hum Mol Genet.

[CR82] Seto ML, Hing AV, Chang J (2007). Isolated sagittal and coronal craniosynostosis associated with TWIST box mutations. Am J Med Genet A.

[CR83] Singh R, Arora R, Kumar R (2018). Clinical notes on chiari malformation. J Craniofac Surg.

[CR84] Speer MC, Enterline DS, Mehltretter L (2003). Chiari type I malformation with or without syringomyelia prevalence and genetics. J Genet Counsel.

[CR85] Swahari V, West AE (2019). Histone demethylases in neuronal differentiation, plasticity, and disease. Curr Opin Neurobiol.

[CR86] Tam SKP, Brodbelt A, Bolognese PA (2020). Posterior fossa decompression with duraplasty in Chiari malformation type 1: a systematic review and meta-analysis. Acta Neurochir.

[CR87] Tatton-Brown K, Douglas J, Coleman K (2005). Genotype-phenotype associations in Sotos syndrome: an analysis of 266 individuals with NSD1 aberrations. Am J Hum Genet.

[CR88] Tian T, Lei Y, Chen Y (2020). Somatic mutations in planar cell polarity genes in neural tissue from human fetuses with neural tube defects. Hum Genet.

[CR89] Tubbs RS, McGirt MJ, Oakes WJ (2003). Surgical experience in 130 pediatric patients with Chiari I malformations. J Neurosurg.

[CR90] Tubbs RS, Lyerly MJ, Loukas M (2007). The pediatric Chiari I malformation: a review. Childs Nerv Syst.

[CR91] Tubbs RS, Beckman J, Naftel RP (2011). Institutional experience with 500 cases of surgically treated pediatric Chiari malformation type I. J Neurosurg Pediatr.

[CR92] Türkmen S, Gillessen-Kaesbach G, Meinecke P (2003). Mutations in NSD1 are responsible for Sotos syndrome, but are not a frequent finding in other overgrowth phenotypes. Eur J Hum Genet.

[CR93] Turski ML, Vidwans SJ, Janku F (2016). Genomically driven tumors and actionability across histologies: BRAF-mutant cancers as a paradigm. Mol Cancer Ther.

[CR94] Twigg SR, Wilkie AOM (2015). A genetic-pathophysiological framework for craniosynostosis. Am J Hum Genet.

[CR95] Twigg SR, Vorgia E, McGowan SJ (2013). Reduced dosage of ERF causes complex craniosynostosis in humans and mice and links ERK1/2 signaling to regulation of osteogenesis. Nat Genet.

[CR96] Twigg SR, Forecki J, Goos JA (2015). Gain-of-function mutations in ZIC1 are associated with coronal craniosynostosis and learning disability. Am J Hum Genet.

[CR97] van Rij MC, Hollink IHIM (2018). Two novel cases expanding the phenotype of SETD2-related overgrowth syndrome. Am J Med Genet A.

[CR98] Vangipuram M, Ting D, Kim S (2013). Skin punch biopsy explant culture for derivation of primary human fibroblasts. J Vis Exp..

[CR99] Wilkie AOM, Johnson D, Wall SA (2017). Clinical genetics of craniosynostosis. Curr Opin Pediatr.

[CR100] Wright CF, Prigmore E, Rajan D (2019). Clinically-relevant postzygotic mosaicism in parents and children with developmental disorders in trio exome sequencing data. Nat Commun.

[CR101] Xu Q, Xiang Y, Wang Q (2019). SETD2 regulates the maternal epigenome, genomic imprinting and embryonic development. Nat Genet.

[CR102] Yamada A, Shimura C, Shinkai Y (2018). Biochemical validation of EHMT1 missense mutations in Kleefstra syndrome. J Hum Genet.

[CR103] Yan K, Rousseau J, Machol K (2020). Deficient histone H3 propionylation by BRPF1-KAT6 complexes in neurodevelopmental disorders and cancer. Sci Adv..

[CR104] Yang F, Sun L, Li Q (2012). SET8 promotes epithelial-mesenchymal transition and confers TWIST dual transcriptional activities. EMBO J.

[CR105] Zhang W, Taylor SP, Ennis HA (2018). Expanding the genetic architecture and phenotypic spectrum in the skeletal ciliopathies. Hum Mutat.

