# Supplementary material for: Chiari 1 malformation and exome sequencing in 51 trios: the emerging role of rare missense variants in chromatin-remodeling genes
Source: Hum Genet. 2020 Dec 18;140(4):625–47. doi: 10.1007/s00439-020-02231-6 (PMC7981314; doi:10.1007/s00439-020-02231-6)
Supplement: Supplementary file 1 — (DOCX 43 kb) [file 439_2020_2231_MOESM1_ESM.docx]

SUPPLEMENTAL METHODS

**Table of Content**

Recruitment and phenotyping of patients

The study was approved by the Ethics Committee of the Meyer Children’s Hospital of Florence. The parents or the affected individual provided written informed consent. The risks and benefits of a research-based whole exome analysis were discussed with each family and an option for disclosure of medically actionable incidental findings provided.

We enrolled patients with either isolated or syndromic C1M. All patients have been evaluated by experts of neurosurgery, radiology as well as clinical genetics. The parents were regularly examined both from a clinical point of view and genetic investigations. Patients had a confirmed diagnosis of C1M on the following criteria: a) caudal displacement of the cerebellar tonsils between 3–5 mm to the plane of the foramen magnum; b) bulbar kinking (cases 13, 22, 23, 26, 35, 37, 45; 13,7 % of cases; c) Klippel–Feil deformity (case 51; 1,96% of cases); d) scoliosis (cases 1, 16, 25, 38, 50; 9,8% of cases); f) hydrocephalus (cases 4, 26; 3,9% of cases). A measurement protocol defining the type of CM was followed upon classical guidelines recommendations (Hidalgo 2020). Supplementary Table 1 (Supplementary Table 1) outlines these standard diagnostic imaging protocols. We have also considered the enviromental factors previously reported as susceptibility factor of C1M as shown in the Supplementary Table 2 (Supplementary Table 2). The correlation between brain anomalies and child, and maternal factors proposed as related to C1M are reported.

The most common symptom in our cases was headache. It occurred in approximately 70% of patients (and in some of the parents carrying the same candidate variant of the proband) and was often localized to the occiput or upper cervical region. This pain was generally exacerbated by Valsalva maneuvers such as laughing, coughing, or sneezing. Scoliosis was present in five cases, being left thoracic and associated to syringomyelia in three of them (1, 25 and 38). Some of the scoliotic patients referred abnormal abdominal reflexes or diffuse pain in the flank or back. Symptoms common in Chiari malformations, as extra-ocular muscle changes, tongue atrophy, down-beat-nystagmus, sleep apnea, and dysphagia, were reported in some of the cases. Moreover, cases 4, 15, 36 and 51 presented torticollis, cases 12 and 31 tics, case 29 dyslexia. Case 26 showed two seizure episodes. Sleep apnea was reported in case 37 and in case 3, the latter having a del(22q11.2) in which this feature is frequent. All cases underwent magnetic resonance imaging (MRI) of the cervical spinal cord to measure the size of the posterior cranial fossa (PCF). If the candidate variant(s) resulted inherited, both the carrier and the non-carrier parent underwent brain MRI, regardless of whether or not they had even minimal signs of disease (MRI images avaible on request).

Supplementary Table 1. *Standard diagnostic imaging protocols*

| Study | Utility |
| --- | --- |
| MR imaging of brain with/without contrast  (axial, sagittal, and coronal) | Measure tonsillar descent. Exclude intracranial/skull-based pathology or anomalies |
| MR imaging of spinal axis with/without contrast (axial and sagittal) | Measure tonsillar descent. Identify presence of syrinx. Exclude skull-based/spinal/skeletal pathology or anomalies |
| X-ray of spinal axis | Evaluate for vertebral anomalies and scoliosis |

Supplementary Table 2 *Signs and factors reported as predisposing to abnormal brain MRI*

| **Factor** | **In detail** | **Cases** |
| --- | --- | --- |
| Craniosynostoses | Sagittal  Coronal  Metopic  Complex | 5, 20, 34, 35  3  18, 38  4 |
| Maternal factors | Mother using epilepsy drug  Mother using antidepressants  Mother using alcohol and or drugs  Maternal diabetes mellitus  Gestational diabetes mellitus  Pre-eclaampsy | None  None  None  None  None  None |
| Child related factors | Gender  Prematurity (<32 weeks -1500 g)  Childs ricketsia  Twin | 21 Male vs 30 Female  None  None  None |

**Brain Magnetic Resonance**

An analysis of the MRI of children and parents from Pediatric Radiology Unit of the Meyer Children’s Hospital was conducted. Syndromic and non-syndromic patients with C1M suspicion and/or craniosynostosis were examined using brain MRI and operated on in our department. All were Caucasian, except for case 24. Brain-MRI was included in our routine clinical protocol and none of our patients was left without imaging since all parents complied with this protocol. For this specific study, after WES analysis, also both parents (and in selected cases the siblings as well as some relatives) underwent brain magnetic resonance with blind procedure. In other words, the operator did not know in which of the two we had identified the same candidate variant present in C1M proband. An experienced paediatric neuroradiologist evaluated all the MR images. Sagittal imaging is generally the best plan to evaluate the presence of C1M: the tonsils are pointed rather than rounded and the furrows are oriented vertically. Axial images through the foramen have shown in all our cases the crowding of the marrow by the tonsils. In some patients we were able to see the syringe in the spinal cord.

Case 5, 20, 34 and 35 (7,8%) were found to have synostosis of the sagittal suture, case 3 (1,96%) had coronal synostosis, case 18 and 38 (3,9%) had metopic and no cases had lambdoid synostosis. Case 4 showed complex craniostenosis (1,96%). In the remaing cases, no craniosynostosis was detected.

In the Supplementary Table 3 (STable 3) brain anomalies identified by MRI in patients with syndromic and non-syndromic C1M based on literature data are reported.

Supplementary Table 3. *Brain anomalies reported in C1M patients* and present/absent in our cases*

| **Anatomical region** | **Brain anomalies** | | **Cases** |
| --- | --- | --- | --- |
| Sellar and parasellar region | Ectopic neurohypophysis, Opticus atrophy with no septal defect | | None |
|  | J-sella | | None |
|  | Empty sella | | None |
| Skull base | Chiari I malformation | congenital hydrocephalus | 4, 26 |
|  |  | cervicomedullary kinking | 13, 22, 23, 26, 35, 37, 45 |
|  |  | focal cerebral heterotopia with epilepsy | None |
| Center of the brain | Agenesis or partial agenesis of corpus callosum | | None |
|  | Joubert anomaly | | None |
|  | Hypoplastic brain stem | | None |
|  | Holoprosencephaly | | None |
|  | Subcortical dysplasia | | None |

***** According to: McClugage and Oakes, 2019; Kular and Cascella, 2020

**Treatment and decompressive surgery**

Our cases with more severe symptoms have undergone surgery for decompression of the cervicomedullary junction and restoration of normal flow of cerebrospinal fluid in the region of the foramen magnum.

Patients were placed in a prone position with slight neck flexion in order to allow visualization of the occipital bone. A midline vertical incision was made from just inferior to the union at level C3. Myofascial dissection was performed along the median raphe. Special attention was given in avoiding muscle dissection from C2 level (semispinalis cervicis and multifidus muscle) to prevent cervical instability and reduce post-operative pain in the neck. Then, a suboccipital craniectomy was performed. The dimensions of the craniectomy varied from 2x2 cm to 3.5x3.5 cm.

Exome Sequencing

From the group of our C1M patients, we could obtain material and clinical information in 51 trios.

*DNA library preparation*

To construct DNA libraries, we used a strategy based on enzymatic fragmentation to produce dsDNA fragments followed by End repair, A-tailing, adapter ligation and library amplification (Kapa Biosystems, Wilmington, MA). Libraries were hybridized with the protocol SeqCap EZ Exome v3 (Nimblegen, Roche, Basel, Switzerland), and sequenced by NextSeq550 (Illumina Inc., San Diego, CA).

*Assembly, Variant Calling*

The reads were aligned with the human reference hg19 genome using Burrows-Wheeler Aligner (BWA), mapped and analyzed with the IGV software (Integrative Genome Viewer, 2013 Broad Institute). The variant call for identification of nucleotide variants was performed using automated *in house* pipelines (Genome Analysis ToolKit Unified Genotyper Module, GATK), for each patient we estimated the average depth of coverage of every captured region.

Validate variants were classified as pathogenic, likely pathogenic, variant of uncertain clinical significance (VUS), likely bening and bening in agreement with the interpretation guidelines of the American College of Medical Genetics and Genomics (ACMG) (Nykamp 2017).

*Variant priorization strategy & bioinformatics pipeline*

Variants were assessed for all 51 individuals and family members based on different modes of inheritance (*de-novo*, autosomal dominant, autosomal recessive and X-linked), facilitated by our *in-house* Next Generation Sequencing Variant Analyzer tool.

In details, we selected only non-synonymous, short insertion/deletion or splice-site variants (30 bp splice acceptor, 30 bp splice donor) with the following characteristics:

- variants with a minor allele frequency ≤ 2% in population databases “1000 Genomes Project” (The 1000 Genomes Project Consortium (2015), “Exome Variant Server” (http://evs.gs.washington.edu/EVS/), dbSNP147 (www.ncbi.nlm.nih.gov/projects/SNP/snp_summary.cgi?view+summary=view+summary&build_id=147), ExAC (Lek 2016), gnomAD (Karczewski 2020). Variants were considered candidate according to the following criteria:

- at least 4 over 6 *in silico* tools (Polyphen-2, SIFT, Mutation Taster, FATHMM, FATHMM MKL, Mutation Assessor; Ng 2006; Adzhubei 2010; Hashem 2013; Schwarz 2014; Kleinberger 2016) provided a damaging score.

- the variant segregated with the disease within the family or was de novo in the patient.

- different cut-off values for the ratio of the reads between the variant and the reference allele were used. Variants, were considered homozygous when at least 80% of the reads was with the variant allele. Variants were considered heterozygous only when at least 30% of the reads was with the variant allele. We considered a variant as X-linked when in a male at least 80% of the reads were with the variant. Other candidate variants were inspected with the Integrative Genomics Viewer to rule out sequencing errors in repetitive regions.

Moreover, to investigate whether C1M could also be an oligogenic disorder, we investigated whether the candidate variants present in the same patient belonged to genes involved in the same pathway.

*Variant validation*

All significative variants were validated by Sanger sequencing. Splice site variants have been evaluated using *in silico* prediction (BGDP http://www.fruitfly.org/).

**Interpretation of variants**

*Application of ACMG criteria*

For the interpretation of the pathogenicity of variants, we applied the recommendation for the interpretation of sequence variants of the ACMG (Nykamp 2017). As databases for previously reported disease-causing mutations, we used ClinVar and the Human Gene Mutation Database (HGMD) Professional (http://www.hgmd.cf.ac.uk/ac/index.php) updated 2020. PS2 was fulfilled for all our *de novo* variants as we confirmed maternity and paternity, based on exome data from trios. PS4 was considered fulfilled if the odds ratio was higher than 5, when comparing the frequency in our patients to the frequency in a control cohort of about 300 controls (*in house* database). PM2 was interpreted according to the frequency of the variant in ExAC and was fulfilled for a variant rare or with a low-frequency if recessive. PP1 was fulfilled if at least one further family member was affected from the disease and carried the variant. BA1, BP2 and BP7 were never fulfilled according to our variant selection criteria. PP3 was fulfilled if at least four out of six *in silico* prediction programmes (SIFT, PolyPhen-2, MutationTaster and MutationAssessor) predicted to be deleterious (i.e. BP4 was not fulfilled). As variants were excluded if an equally affected family member did not carry the same mutation or if an unaffected family member also carries the same mutation, BS4 was never fulfilled. The final categorization based on these criteria was then performed on the flowchart described in the Supplementary Fig 1 (SFig1).

**CGH-Array**

CGH-array was performed using a custom Agilent Human Genome CGH Microarray (Agilent Technologies, Santa Clara, CA). We used the same protocol as previously described (Palazzo 2017). This platform has a resolution of 40 kb in the regions of interest. Text output from the quantitative analyses were imported into Genomic Workbench Standard Edition 5.0 software (Agilent Technologies).

**Total RNA extraction and gene expression profiling by Affymetrix GeneChip analysis**

Gene expression profiling was performed by using the Affymetrix GeneChip technology and GeneChip™ Human Transcriptome Array 2.0 (Affymetrix, Santa Clara, CA) according to manufacturer’s standardized protocols. After the scanning procedure, data files were checked for quality parameters. Microarray data analysis was performed according to Affymetrix suggestions (GeneChip Expression Analysis: data analysis fundamentals in <http://www.affymetrix.com>). Data are *available* on request.

Image and expression data files were generated with Affymetrix MAS (MicroArray Suite) 5.0. Low level and statistical analysis were done using R 2.3. Microarray data were first processed in R environment http://www.r-project.org by Affymetrix package to identify present/absent probe set, and then subjected to a normalization step. We normalized data according to MAS method: background correction with MAS method, normalization at a probe level with constant method (a global adjustment by a constant value to equalize the chip-wide mean signal intensity between chips) (Harr 2006).

In order to identify differentially expressed genes, at statistically significant level, we applied a t-statistic variant approach. We used the significance analysis of microarrays (SAM) method (Boareto 2014), in which the t-statistic has a constant value added to the standard deviation. We performed SAM analysis with Siggenes package (Schwender 2020). All the analysis was written in the freely available statistical language R.

In order to evaluate whether and what pathways, biological processes, molecular functions were modulated by *SETD* family variants, global expression profiling of RNA obtained by fibroblasts of probands (cases 23, 25, 28), carrier familial members and four male controls with a mean age of 12,5 years was evaluated. No differences were observed by SAM analysis according to the following comparisons:

Controls vs Chiari probands’ RNA fibroblasts

Chiari probands’ vs parent’s RNA fibroblasts (cases 25 vs affected father, 28 vs affected mother). The parents of case 23 did not agree to their cutaneous biopsy.

Control vs Chiari probands’ and parents’ RNA fibroblasts

**Legend to Supplementary Figures**

**Supplementary Fig1.** Flowchart illustrating filtering process and variants selection used to identify pathogenic variations.

**Supplementary Fig2.** Schematic representation of SETD2 protein with the location of functional domains and variants present both in our cases and those reported in the literature. The seven domains are indicated with boxes: AWS domain (light blue), SET domain (fuchsia), post-SET domain (yellow), Asp-B-Hydro-N domain (green), Low charge region domain (orange), WW domain (magenta) and SRI domain (purple). In red all SETD2 variants identified in our cases with C1M; in purple *SETD2* variants reported by Luscan A et al. (Luscan 2014); in blue, green, black, and brown, *SETD2* variants reported with syndromic severe ID (Lumish 2015; Tlemsani 2016; Faundes 2018; van Rij 2018; Aldinger 2019). No correlation is detectable between variants localization and clinical manifestation.

**AWS**: associate with SET domain; **Asp-B-Hydro-N**: Aspartyl beta hydroxylase N terminal region; **SRI**: Set2 Rpb1 interacting domain

**Supplementary Fig3.**

a. Schematic representation of SETD1B protein with the location of functional domains and variants present both in our cases and those reported in the literature. The five domains are indicated with boxes: RRM domain (light blue), Trypan_PARP domain (orange), N-SET domain (green), SET domain (fuchsia) and post- SET domain (yellow). In red all *SETD1B* variants identified in our cases with C1M; in blu and in purple *SETD1B* variants previously reported (Hiraide 2018; Krzyzewska 2019). Most of the patients with syndromic ID have variants within one of the SETD1B domains in contrast to those detected in our cohort.

**RRM**: RNA recognition motif in vertebrate histone-lysine; **PARP**: procyclic acidic ripetitive protein

b. Schematic representation of deletions, based on UCSC Genome Browser (https://genome.ucsc.edu/cgi-bin/hgGateway) with refseq genes using hg 19, in published cases that included the SETD1B gene (Chouery 2013; Qiao 2013; Palumbo 2015; Labonne 2016). The pink area represents the deletion in each patient. In the box red area is included the SETD1B gene.

**Supplementary Fig4.** Schematic representation of NSD3 protein with the location of functional domains and the variant present in case 19. The nine domains are indicated with boxes: PWWP 1-2 (green), PHD1-5 domain (orange), AWS domain (fuchsia), SET domain (purple). In red the variant identified in our case 19. Multiple amino acid sequences from different species using Cobalt tool from NCBI (Papadopoulos 2007) are showed in the bottom.

Red highlight indicates the amino acid residue conserved amongst different species.

**Supplementary Fig5.** Schematic representation of KMT2E protein with the location of functional domains and variants present in our case. The five domains are indicated with boxes: HCFC1 domain (light yellow), PHD_MLL5 domain (light blue), SET domain (fuchsia), ARGLU domain (dark orange) and Med15 domain (green). In red, variants identified in our case 35 in compound heterozygosity.

Multiple amino acid sequences from different species using Cobalt tool from NCBI (Papadopoulos 2007) are shown in the bottom.

Red highlight indicates the amino acid residue conserved amongst different species.

**Supplementary Fig6.** Schematic representation of KDM5B and KDM6B proteins with the location of functional domains and variants present in our cases.

a. KDM5B protein: the eight domains are indicated with boxes: JmjN domain (light blu), BRIGHT domain (purple), PHD1 domain (grey), JmjC domanin (fuchsia), Zf-C5HC2 (yellow), PLU1 domain (green), PHD2 (orange), PHD3 (blue).

b. KDM6B protein: the two domains are indicated with boxes: Mito_fiss domain (light yellow), Jmjc domain (blue).

In red, variants identified in our cases.

Multiple amino acid sequences from different species using Cobalt tool from NCBI (Papadopoulos 2007) are shown in the bottom.

Red highlight indicates the amino acid residue conserved amongst different species.

Supplementary References

Adzhubei IA, Schmidt S, Peshkin L, et al. (2010) A method and server for predicting damaging missense mutations. Nat Methods.7:248-249. https://doi: 10.1038/nmeth0410-248

Aldinger KA, Timms AE, Thomson Z, et al. (2019) Redefining the Etiologic Landscape of Cerebellar Malformations. Am J Hum Genet. Sep;105:606-615. doi: 10.1016/j.ajhg.2019.07.019

Boareto M, Catich N. (2014) t-Test at the Probe Level: An Alternative Method to Identify Statistically Significant Genes for Microarray Data. Microarrays (Basel) 3:340-35. https://doi: 10.3390/microarrays3040340

Chouery E, Choucair N, Abou Ghoch J, et al. (2013) Report on a patient with a 12q24.31 microdeletion inherited from an insulin-dependent diabetes mellitus father Mol Syndromol Mar.4:136-42. doi: 10.1159/000346473

Faundes V, Newman WG, Bernardini L, et al. (2018) Histone Lysine Methylases and Demethylases in the Landscape of Human Developmental Disorders. Am J Hum Genet. Jan;102:175-187. doi: 10.1016/j.ajhg.2017.11.013

Harr B and Schlötterer C. (2006) Comparison of algorithms for the analysis of Affymetrix microarray data as evaluated by co-expression of genes in known operons. Nucleic Acids Res. 34(2) https://doi:10.1093/nar/gnj010

Hashem A Shihab, Julian Gough, David N Cooper, et al. (2013) Predicting the Functional, Molecular, and Phenotypic Consequences of Amino Acid Substitutions using Hidden Markov Models. Hum Mutat. 34:57-65. https://doi: 10.1002/humu.22225

Hidalgo JA, Varacallo M. (2020) Arnold Chiari Malformation. StatPearls. Treasure Island (FL): StatPearls Publishing. https://www.ncbi.nlm.nih.gov/books/NBK554609/

Hiraide T, Nakashima M, Yamoto K, et al. (2018) De novo variants in SETD1B are associated with intellectual disability, epilepsy and autism. Hum Genet.137:95-104. doi: 10.1002/epi4.12339

Karczewski K.J., Francioli L.C., Tiao G. et al. (2020) The mutational constraint spectrum quantified from variation in 141,456 humans. Nature 581:434–443. https://doi.org/10.1038/s41586-020-2308-7

Kleinberger J, Maloney KA, Pollin TI, et al. (2016) An openly available online tool for implementing the ACMG/AMP standards and guidelines for the interpretation of sequence variants. Genet Med 18: 1165. https://doi:10.1038/gim.2016.13

Krzyzewska IM, Maas SM, Henneman P, et al. (2019) A genome-wide DNA methylation signature for SETD1B-related syndrome. Clin Epigenetics. 11:156. doi: 10.1186/s13148-019-0749-3

Labonne JDJ, Lee KH, Iwase S, et al. (2016) An atypical 12q24.31 microdeletion implicates six genes including a histone demethylase KDM2B and a histone methyltransferase SETD1B in syndromic intellectual disability. Hum Genet;135:757-71 doi: 10.1007/s00439-016-1668-4

Lek M, Karczewski KJ, Minikel EV et al. (2016) Analysis of protein-coding genetic variation in 60,706 humans. Nature. 536:285-91. https://doi: 10.1038/nature19057

Lumish HS, Wynn J, Devinsky O, Chung WK. (2015) Brief Report: SETD2 Mutation in a Child with Autism, Intellectual Disabilities and Epilepsy. J Autism Dev Disord Nov;45:3764-70. doi: 10.1007/s10803-015-2484-8

Luscan A, Laurendeau I, Malan V, et al. (2014) Mutations in SETD2 cause a novel overgrowth condition. J Med Genet.Aug;51:512-7 doi: 10.1136/jmedgenet-2014-102402

Ng PC, Henikoff S. (2006) Predicting the effects of amino acid substitutions on protein function. Annual review of genomics and human genetics 7:61-80. https://doi: 10.1146/annurev.genom.7.080505.115630

Nykamp K, Anderson M, Powers M, et al. (2017) Sherloc: a comprehensive refinement of the ACMG-AMP variant classification criteria. Genet Med 19:1105-1117. doi: 10.1038/gim.2017.37. AND (2020) Genet Med. 22:240. doi: 10.1038/s41436-019-0624-9

Palazzo V, Provenzano A, Becherucci F, et al. (2017) The genetic and clinical spectrum of a large cohort of patients with distal renal tubular acidosis. Kidney Int. 91:1243-1255. doi: 10.1016/j.kint.2016.12.017

Palumbo O, Palumbo P, Delvecchio M, et al. (2015) Microdeletion of 12q24.31: report of a girl with intellectual disability, stereotypies, seizures and facial dysmorphisms. Am J Med Genet A;167:438-44. doi: 10.1002/ajmg.a.36872

Papadopoulos JS, Richa Agarwala (2007) COBALT: constraint-based alignment tool for multiple protein sequences. Bioinformatics May;23:1073-9. doi: 10.1093/bioinformatics/btm076

Qiao Y, Tyson C, Hrynchak M, et al. (2013) Clinical application of 2.7M Cytogenetics array for CNV detection in subjects with idiopathic autism and/or intellectual disability. Clin Genet Feb;83:145-54. doi: 10.1111/j.1399-0004.2012.01860.x

Schwarz JM, Cooper DN, Schuelke M, et al. (2014) MutationTaster2: mutation prediction for the deep-sequencing age. Nature methods 11:361-362. https://doi: 10.1038/nmeth.2890

Schwender H (2020). siggenes: Multiple Testing using SAM and Efron's Empirical Bayes Approaches. R package version 1.62.0. www.bioconductor.org https://doi: 10.18129/B9.bioc.siggenes

The 1000 Genomes Project Consortium (2015) A global reference for human genetic variation. Nature. 526:68-74. https://doi:10.1038/nature15393

Tlemsani C, Luscan A, Leulliot N, et al. (2016) SETD2 and DNMT3A screen in the Sotos-like syndrome French cohort. J Med Genet Nov;53:743-751. doi: 10.1136/jmedgenet-2015-103638

van Rij M, Hollink IHI, Terhal PA, et al. (2018) Two novel cases expanding the phenotype of SETD2-related overgrowth syndrome. J Med Genet A May;176:1212-1215. doi: 10.1002/ajmg.a.38666
