# Supplementary figures and images for: Chiari 1 malformation and exome sequencing in 51 trios: the emerging role of rare missense variants in chromatin-remodeling genes
Source: Hum Genet. 2020 Dec 18;140(4):625–47. doi: 10.1007/s00439-020-02231-6 (PMC7981314; doi:10.1007/s00439-020-02231-6)

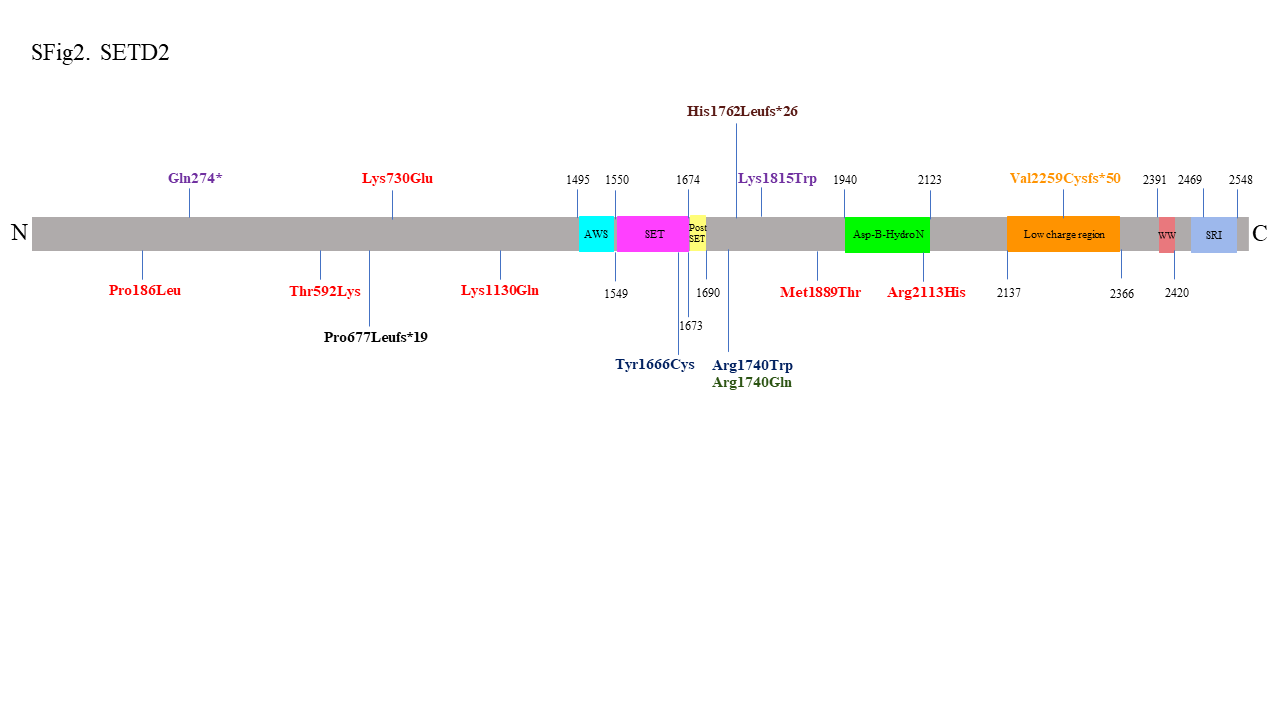

Supplement: Supplementary file 3 — (TIF 103 kb) [file 439_2020_2231_MOESM3_ESM.tif]

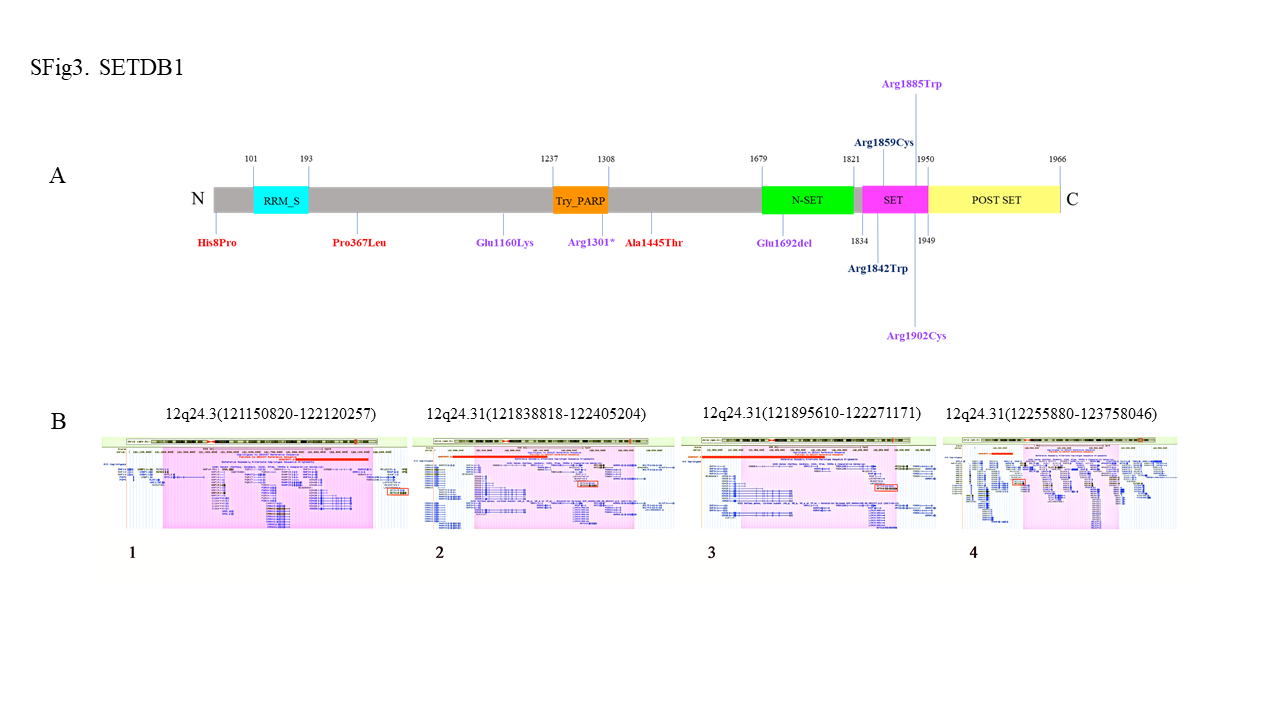

Supplement: Supplementary file 4 — (TIF 330 kb) [file 439_2020_2231_MOESM4_ESM.tif]

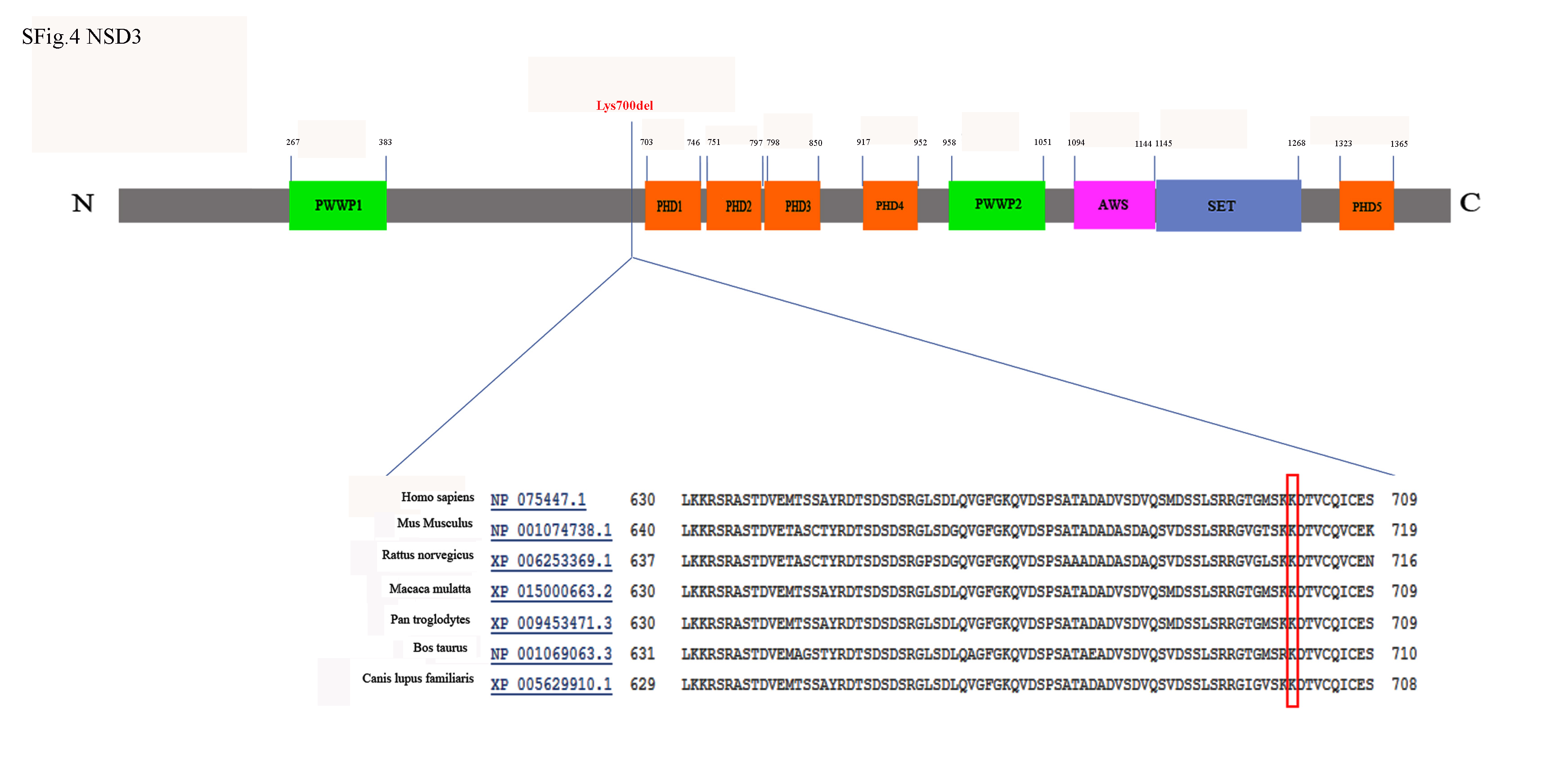

Supplement: Supplementary file 5 — (TIF 17518 kb) [file 439_2020_2231_MOESM5_ESM.tif]

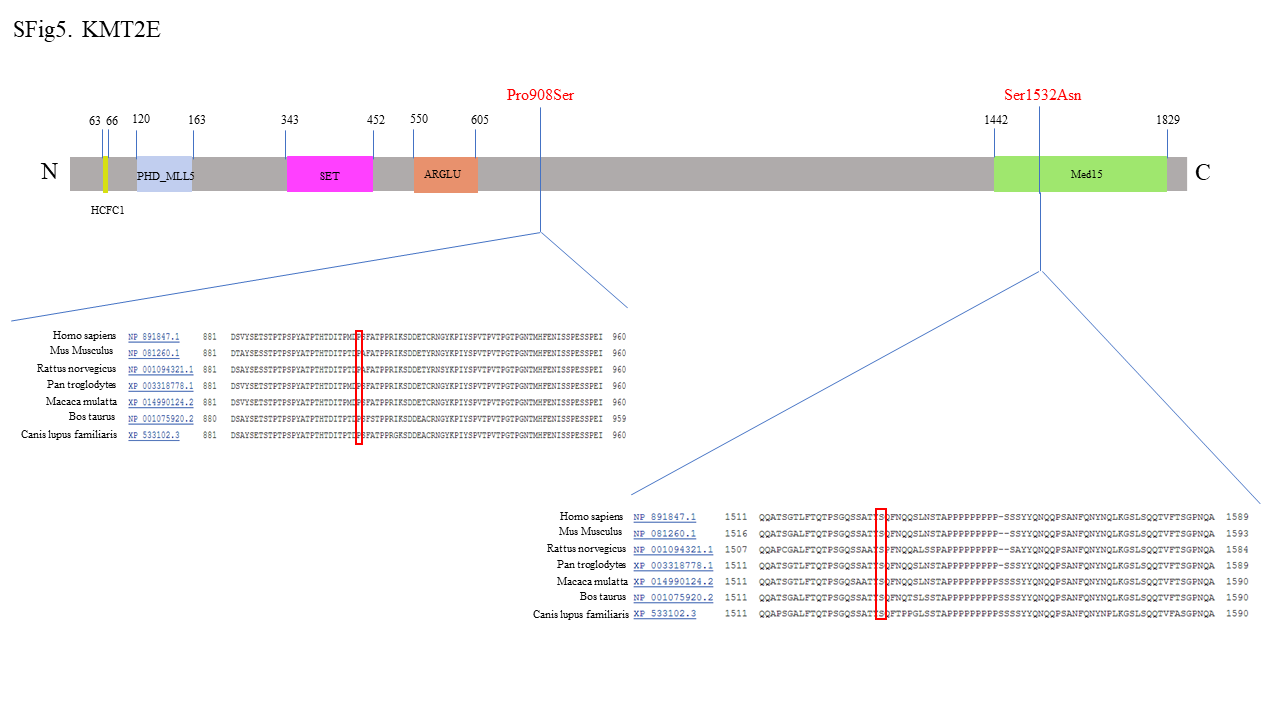

Supplement: Supplementary file 6 — (TIF 307 kb) [file 439_2020_2231_MOESM6_ESM.tif]

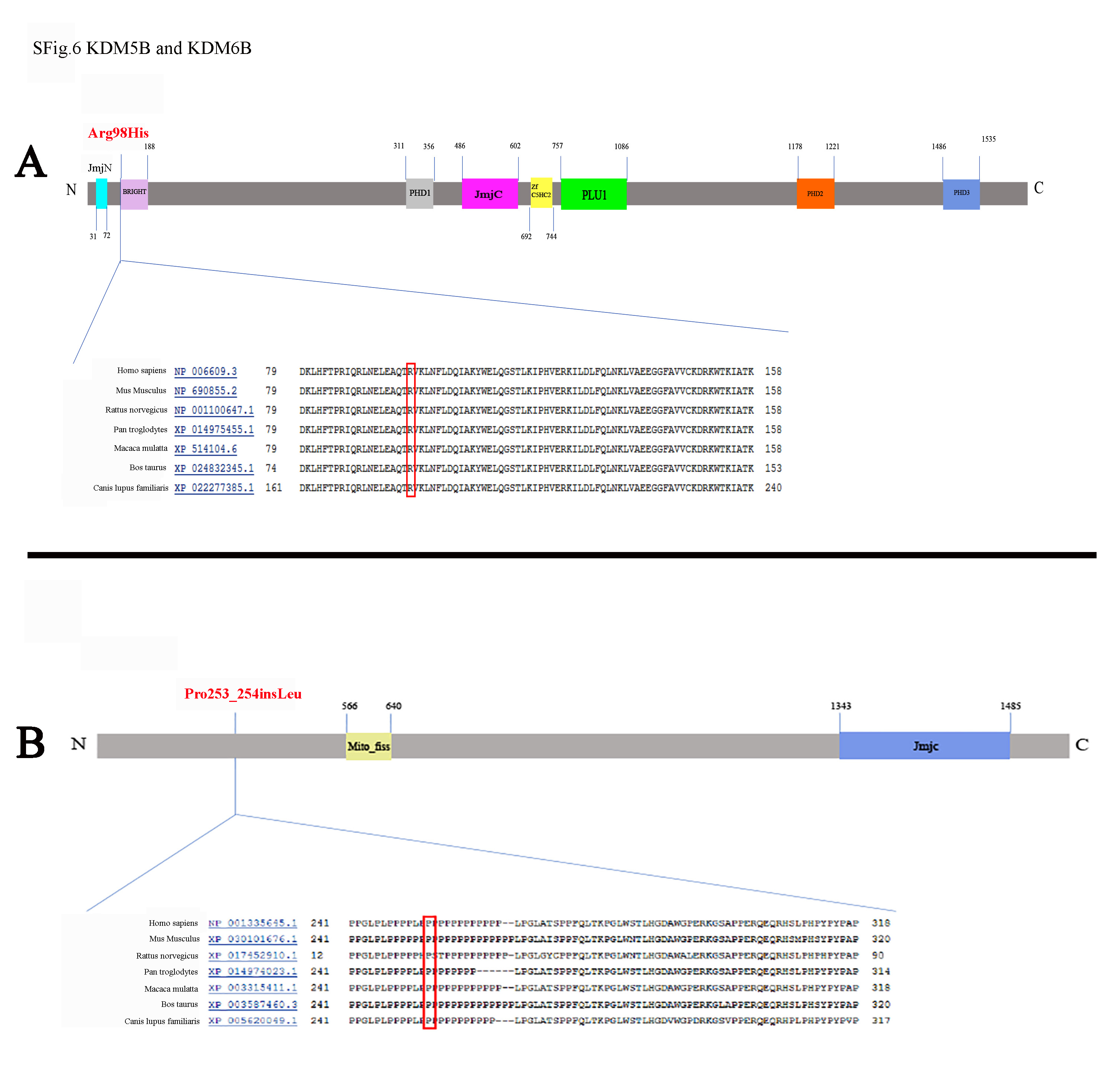

Supplement: Supplementary file 7 — (TIF 25831 kb) [file 439_2020_2231_MOESM7_ESM.tif]
